# The Effectiveness of Wearable Electronic Device System–Supported Physical Activity Programs for Cancer Survivors: Meta-Analysis of Randomized Controlled Trials

**DOI:** 10.2196/74347

**Published:** 2025-08-14

**Authors:** Zezhang Wang, Yunhuan Li, Qian Wang, Yonglin Su

**Affiliations:** 1 Center of Rehabilitation Medicine, West China Hospital of Sichuan University, No 37, Guoxue Alley, Wuhou District, Chengdu, Sichuan, 610041, China, 86 28 85422847; 2 Rehabilitation Key Laboratory of Sichuan Province, West China Hospital of Sichuan University, Chengdu, Sichuan, China; 3 Department of Nursing, West China Hospital, Sichuan University/West China School of Nursing, Sichuan University, Chengdu, Sichuan, China

**Keywords:** oncology, digital health, physical activity, quality of life, PRISMA

## Abstract

**Background:**

As cancer is increasingly regarded as a chronic disease, it is essential to support cancer survivors’ self-management and enhance their quality of life (QoL). Although a physically active lifestyle can help alleviate symptom burden, improve QoL, and even benefit survival among cancer survivors, many remain physically inactive. Wearable electronic device systems (WEDSs) have become increasingly integrated into daily life and may offer a potential solution to promote physical activity (PA) and improve QoL in this population. However, existing findings remain modest and inconclusive.

**Objective:**

This meta-analysis aims to evaluate (1) the effects of WEDS-supported PA programs on improving PA, sedentary behavior, BMI, and QoL in cancer survivors; and (2) the effects of various types of these interventions.

**Methods:**

A comprehensive literature search was conducted across PubMed, Embase, Web of Science, CENTRAL, and MEDLINE from database inception through July 31, 2024. Two authors independently screened the articles, extracted the data, and evaluated the methodological quality of the included studies using the Cochrane Risk-of-Bias tool 2. Data synthesis was performed using R Studio. The effects of the interventions were determined by calculating standard mean differences (SMDs) and 95% CIs, while heterogeneity was assessed using *I*² statistics and *P* values. Subgroup analysis was conducted to assess whether the effects differed by the formats of the partnering tools and the duration of the intervention. Sensitivity analysis was performed using the one-study-out method to evaluate the robustness of the results, and the Egger test was conducted to assess small study effects. Statistical significance for the overall effect was considered when the 2-tailed *P* value was less than .05.

**Results:**

A total of 46 randomized controlled trials, involving 3727 patients, were included in this meta-analysis. The results indicated that WEDS-supported PA programs significantly improved objectively measured moderate-to-vigorous-intensity physical activity (MVPA; SMD 0.66, 95% CI 0.47-0.86, *P*<.001, *I*^2^=69%), subjectively reported PA (SMD 0.5, 95% CI 0.23-0.77, *P*<.001, *I*^2^=79%), steps per day (SMD 0.5, 95% CI 0.23-0.77, *P*=.009, *I*^2^=79%), and QoL (SMD 0.19, 95% CI 0.08-0.31, *P*<.001, *I*^2^=33%) among cancer survivors. Subgroup analysis revealed that interventions incorporating multipartnering tools (no fewer than 2 formats) were effective in improving subjectively reported PA, steps per day, and QoL. Long-term interventions (≥12 weeks) improved objectively measured MVPA, subjectively reported PA, steps per day, and QoL. Interventions tailored to specific cancer types significantly improved steps per day (SMD 0.59, 95% CI 0.1-1.08, *P*=.008, *I*^2^=83%) and QoL (SMD 0.14, 95% CI 0.04-0.23, *P*=.006, *I*^2^=0%).

**Conclusions:**

We observed that WEDS-supported PA programs are effective in improving the level of PA (both objectively and subjectively), steps per day, and QoL among cancer survivors, but showed no significant effects on sedentary behavior or BMI. In the future, the use of multipartnering tools, appropriate intervention duration, and tailored PA programs should be carefully considered when developing WEDS-supported PA interventions. Further promotion and refinement of WEDS-supported PA programs are warranted.

## Introduction

Owing to advancements in early detection and breakthroughs in cancer treatment, the number of long-term cancer survivors has significantly increased, transforming cancer into a chronic condition and making it a growing global public health concern [1]. When cancer is considered a chronic disease, it is essential to support cancer survivors’ self-management and enhance their quality of life (QoL). However, cancer survivors often experience various symptom burdens, including reduced cardiorespiratory fitness and muscle strength, fatigue, sleep disturbances, and emotional distress, all of which affect their QoL and mortality [1-8]. These symptom burdens and decreased QoL are even associated with patient survival [9-12]. Maintaining physical activity (PA) has emerged as a promising lifestyle approach for cancer survivors. PA refers to any bodily movement produced by skeletal muscles that requires energy expenditure [13]. For cancer survivors, adequate PA, especially moderate-to-vigorous-intensity PA (MVPA), can improve their cardiorespiratory and muscular fitness, alleviate symptom burden (such as cancer-related fatigue), and enhance QoL [14]. However, when cancer survivors are diagnosed with cancer, their engagement in even little or leisure PA significantly decreases, whereas their time spent sitting increases, which negatively impacts their survival [15,16]. Barriers that prevent cancer survivors from participating in PA may include their symptom burden (such as pain, fatigue, and lymphedema), social factors (such as lack of time, motivation, and support from health care professionals [HCPs]), and lack of information (such as recommendations on PA) [17]. Thus, it is essential to identify interventions that can encourage or remind cancer survivors to increase their level of PA and provide support to enhance their physical fitness, such as muscular fitness and cardiorespiratory fitness, ultimately improving their QoL and survival.

Digital health, defined as the use of “digital technologies for health” [18], including mobile health (mHealth) apps, electronic health records, electronic medical records, wearable electronic devices (WEDs), telehealth and telemedicine, and personalized medicine [19], is an influential force in the progression of global health care toward improved accessibility and quality [20]. WEDs refer to any kind of electronic device designed to be worn on the user’s body, as either an accessory or an implant [21]. In the context of behavior change techniques [22], interventions supported by WEDs have become increasingly prevalent among cancer survivors to increase their PA by collecting physical and physiological information, enabling continuous real-time self-health surveillance, and providing stimuli for behavior change [23-26]. Moreover, WEDs can be combined with partnering tools to form a wearable electronic device system (WEDS), where partnering tools may include telephone calls, SMS text messages, apps, or websites. Interventions supported by WEDS are effective intervention modalities and can offer an optional and novel approach to promoting PA among cancer survivors [25,27,28].

In WEDS-supported PA programs, HCPs usually set PA goals for participants, WEDs facilitate self-monitoring and data collection, and partnering tools typically enable patient contact with HCPs or provide timely feedback from HCPs (such as consultation, goal resetting, and guidance) [28]. Owing to these benefits and their portability, studies exploring WEDS-supported PA in oncology rehabilitation have surged dramatically over the past decade [23,29,30]. Positive effects have been observed in improving PA among older adults, adults, and patients with diabetes, cardiovascular-related diseases, and chronic obstructive pulmonary disease [31-35]. Although evidence suggests that WEDS-supported PA programs can benefit patients with cancer by increasing PA levels and improving health-related outcomes (such as fatigue, muscular fitness, aerobic fitness, and QoL), findings from existing studies on the effects of these programs remain inconsistent [8,23,29,30]. For example, Singh et al [23] reported that WEDS-supported PA programs led to a statistically significant increase in patients’ daily step counts, whereas Teo et al [30] found no statistically significant difference in daily step counts between the experimental and control groups. Additionally, in other forms of eHealth-supported interventions for cancer survivors, effectiveness varies depending on the type of eHealth and the duration of the intervention. For example, both Li et al [36] and Su et al [37] reported that the effectiveness of internet-based digital health interventions differed across different subgroups based on the format or duration of the intervention. Moreover, previous studies have consistently overlooked the role of partnering tools, and no researcher has investigated which types of partnering tools may better integrate with WEDs to enhance their effectiveness. Additionally, although numerous studies on this topic have been published, most have focused only on specific types of cancer or examined a limited range of health-related outcomes [29,30]. Furthermore, prior research in this area has predominantly focused on examining the feasibility, acceptability, or overall effects of WED-supported PA programs on cancer survivors. To our knowledge, no meta-analysis has focused specifically on WEDS-supported PA programs, nor has any examined subgroup effects on diverse outcomes, such as different formats of partnering tools for improving PA levels, BMI, or QoL, and decreasing sedentary behavior.

Thus, the objectives of this meta-analysis are (1) to evaluate the effects of WEDS-supported PA programs on increasing PA-related outcomes and QoL, and (2) to explore which type of WEDS is most effective and the optimal duration of the intervention for cancer survivors.

## Methods

### Study Design

This meta-analysis was conducted in accordance with the PRISMA (Preferred Reporting Items for Systematic Reviews and Meta-Analyses) 2020 statement [38] and has been duly registered with the International Prospective Register of Systematic Reviews (PROSPERO; registration number CRD42024582905). As the data utilized in this research were exclusively sourced from previously published studies, ethical approval and informed consent were not required.

### Search Strategy

After consulting a professor of statistics, a comprehensive literature search was carried out, covering the period from the inception of the databases to July 31, 2024. The search was conducted across PubMed, Embase, Web of Science, CENTRAL, and MEDLINE, with access to the full text of the articles. The development of the search strategy was guided by the PICOS (Participants, Interventions, Comparisons, Outcomes, and Study Design) framework, along with the guidelines provided by the Cochrane Collaboration to ensure the integrity of the analysis. This strategy incorporated the use of MeSH (Medical Subject Headings) terms, textual keyword searches, and Boolean logic operations, supplemented by keywords from titles or abstracts, including terms such as *neoplasms*, *carcinomas*, *tumors*, *cancer*, *caregivers*, *PA trackers*, *wearable*, *telemedicine*, and *telerehabilitation*. All search strategies used are presented in Multimedia Appendix 1. The search was limited to studies involving humans and randomized controlled trials and was conducted in English. Moreover, we conducted a rigorous manual review of the bibliographies of the retrieved articles to identify and obtain supplementary relevant scholarly works, thereby enhancing the depth of our analytical inquiry.

### Study Eligibility Criteria

This meta-analysis considered studies for inclusion based on the following criteria: (1) participants were survivors of any type of cancer, regardless of sex or cancer stage; (2) patients in the intervention group received WEDS-supported PA programs, which included reminders to change behavior, consultations with HCPs, or social support from other patients; (3) patients in the control group received usual care or were placed on a waitlist; (4) the outcomes included at least one of the following indices—objectively measured MVPA, subjectively reported PA, sedentary behavior, QoL, or BMI—without restrictions on the measures used; (5) the publications were written in English; and (6) the studies were designed as randomized controlled trials. Studies were excluded if they were only registered but not yet conducted or if relevant data were incomplete.

### Study Selection and Data Extraction

The reference management software EndNote X9 (Clarivate Plc) was used to import and screen the titles and abstracts of the studies. Duplications were first removed automatically by EndNote and then meticulously screened by researchers. To ensure alignment with the inclusion criteria, 2 independent authors (ZW and YL) concurrently conducted a thorough screening of the titles and abstracts. Subsequently, they carefully evaluated the full texts of the papers based on the predetermined eligibility criteria. Any discrepancies in the screening process were resolved through discussion or by consulting a third author (QW). Data extraction from the included studies was performed independently by 2 authors (ZW and YL), who meticulously recorded the information using a predefined data extraction template. This template encompassed a range of details, including the first author’s name, year of publication, country where the study was conducted, participants’ ages (mean and SD), sample size, type of cancer diagnosed, types of WEDs and associated tools used, intervention content, duration of the interventions, outcome measures employed, and timing of assessments.

### Quality Assessment

The methodological quality and risk of bias in the included studies were meticulously assessed by 2 independent reviewers, using the Risk of Bias Tool 2, version 5.1.0. A total of 7 domains were evaluated: random sequence generation (selection bias), allocation concealment (selection bias), blinding of participants and personnel (performance bias), blinding of outcome assessment (detection bias), incomplete outcome data (attrition bias), selective reporting (reporting bias), and other potential sources of bias. Each domain was graded as “low risk” of bias, “high risk” of bias, and “unclear risk” of bias. The official Cochrane Excel tool was used to automatically compute the overall risk. Disagreements were resolved through discussion or, when necessary, by consulting a third author.

### Data Synthesis and Analysis

Utilizing R Studio (R Foundation), we conducted heterogeneity evaluations and performed the meta-analysis. To quantify the intervention effects, we computed the standard mean difference (SMD) along with its corresponding 95% CI, and presented the results using forest plots. To obtain more robust results, all data were pooled and analyzed using a random-effects model, while a fixed-effects model was applied when the number of included studies was small (no more than 5) [39]. In cases where a multiarm trial was included, the shared group was divided into subgroups of approximately equal size, 1 for each experimental group [40,41]. In addition, we assessed statistical heterogeneity across all included studies using the *I*^2^ statistic and *P* value. When there were 10 or more studies, the Egger test was conducted to assess small-study effects, with a *P* value below .05 indicating the possible presence of such effects [42]. To evaluate the robustness and reliability of the pooled results, a sensitivity analysis was performed, using the one-study-out method. Statistical significance for the overall effect was established when the 2-tailed *P* value was less than .05.

## Results

### Search Results and Selection

**Figure 1.**
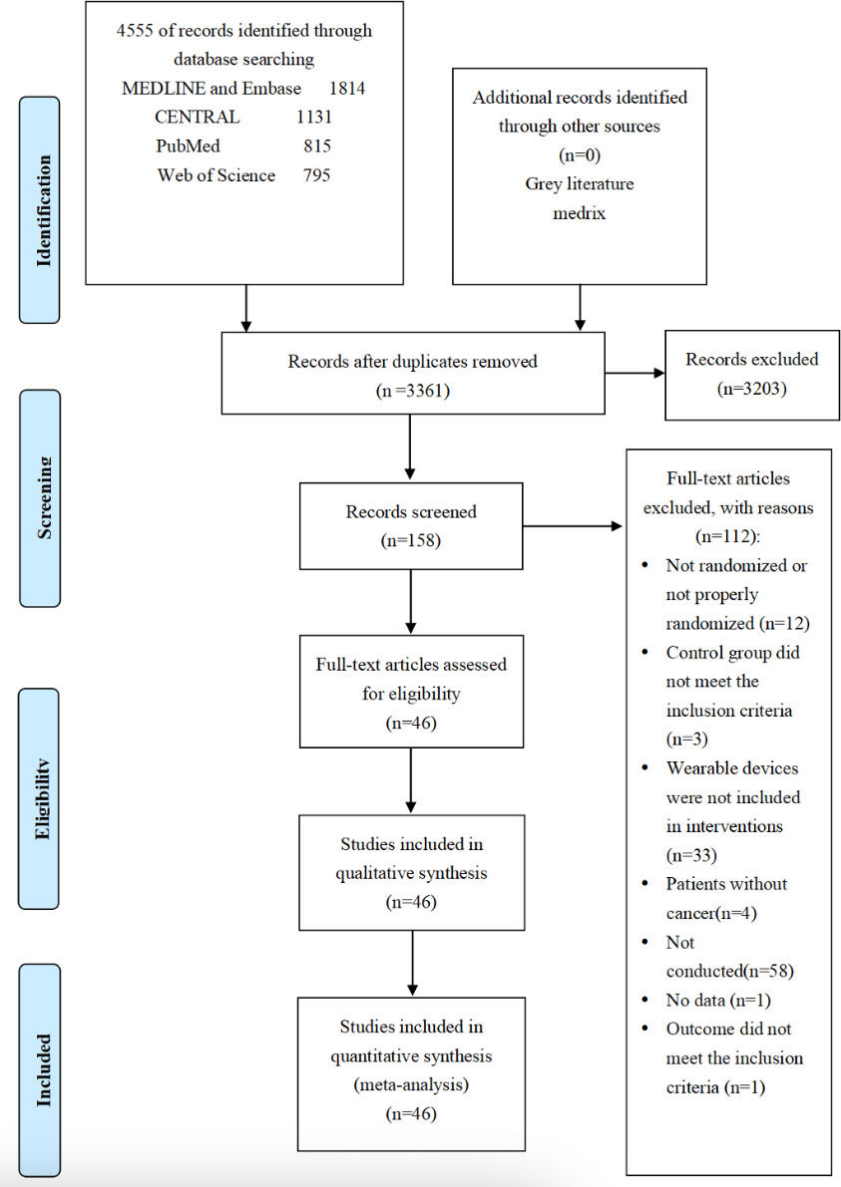
PRISMA (Preferred Reporting Items for Systematic Reviews and Meta-Analyses) flow diagram of the study selection process.

The initial search across 5 electronic databases identified 4555 articles. After 1194 duplicates were removed both automatically and manually, 3361 articles were excluded based on their titles and abstracts. Following this initial screening, the full texts of the remaining 153 articles were retrieved, resulting in a final total of 46 studies included in the meta-analysis. The procedures for search and selection are delineated in Figure 1.

### Description of Included Studies

#### Study Characteristics

The attributes of the 46 studies included in this analysis are presented in Multimedia Appendix 2. All studies, conducted as randomized controlled trials, were published between 2005 and 2024 across 8 countries: the United States of America (31 studies) [43-73], Canada (1 study) [74], the United Kingdom (2 studies) [75,76], New Zealand (1 study) [77], Australia (6 studies) [78-83], the Netherlands (2 studies) [84,85], Korea (2 studies) [86,87] and China (1 study) [88].

#### Characteristics of Cancer Survivors

A total of 3727 cancer survivors were enrolled in the studies, with the number of participants ranging from 11 [65] to 412 [85]. The mean age of the included cancer survivors ranged from 12.7 (SD 7.87) years [53] to 73.79 (SD 7.74) years [65]. Regarding cancer types, 19 studies enrolled participants diagnosed with nonspecific types of cancer [45-47,53,54,56,59,67,72,73,75-80,82,85,88], while 27 focused solely on a single cancer type, including breast cancer (15 studies) [43,44,51,52,57,58,60,61,63,64,66,71,74,81,83], colorectal cancer (6 studies) [48,49,62,68,69,86], prostate cancer (4 studies) [55,65,70,87], leukemia or lymphoma (1 study) [50], and glioma (1 study) [84].

#### Characteristics of WEDS-Supported PA Programs

In the included studies, the intervention duration ranged from 4 weeks [45] to 48 weeks [51], with an average duration of 13.4 weeks. The WEDS-supported PA programs consisted of 2 components: WEDs and partnering tools.

WEDs play a role in step counting, reminders, and data storage. The WEDs used in these studies included pedometers (n=13) [43,56,57,61-63,65,66,75,77,79,85,86], smartwatches (n=4) [64,74,83,84], breath monitors (n=1) [45], smart bands (n=25) [44,46-52,54,55,58,59,67-73,76,78,80-82,87], intelligent sports bracelets (n=1) [88], headbands (n=1) [60], and activity monitors, with no mention of the specific type (n=1) [53]. There are some similarities between smart bands, intelligent sports bracelets, and smartwatches; however, intelligent sports bracelets are considered more fashionable due to their appearance resembling a traditional bracelet, while smart bands are slimmer and simpler in design, focusing primarily on fitness tracking and health monitoring [89]. In comparison, smartwatches have a watch-like form and offer more versatile functionalities, including apps and notifications [90].

Partnering tools in WEDS differ in their functions, including reminders, consultation, education, and data transmission for researchers. The types of partnering tools used included websites/web pages (n=5) [51,53,79,80,85], apps (n=7) [45,50,58,60,64,66,87], telephone calls (n=13) [43,56,57,61-63,65,71,74,75,77,83,88], SMS text messages (n=2) [48,76], or their combinations (n=19) [44,46,47,49,52,54,55,59,67-70,72,73,78,81,82,84,86].

In the 46 included articles, the behavior change techniques used in the interventions included goal setting, self-monitoring, feedback and monitoring, and social support. All interventions used goal setting, self-monitoring, and feedback and monitoring, while 8 studies [44,48,51,55,60,65,68,73] incorporated social support.

#### Characteristics of the Controls

Most of the patients in the control groups received usual care (n=35), which included education from HCPs (n=33) or only access to websites or an app without reminders (n=2) [43,44,47-50,53-56,58-70,72,75,77,78,80-82,85-88]. Others were placed on a waiting list (n=11) to receive the respective interventions after the trials [45,46,51,52,57,71,73,74,79,83,84].

#### Outcome Measures

Outcome measures encompassed a diverse array, with assessments conducted at varying intervals and across different follow-up periods for participants.

#### Objectively Measured Moderate-to-Vigorous-Intensity Physical Activity

Researchers in 20 studies assessed objectively measured MVPA using an ActiGraph accelerometer [44,46,47,50,52-55,58,59,64,67-69,71,72,78,80-82,85]. Anderson et al [75] used a SenseWear PA monitor to assess patients’ objectively measured PA, while Ferrante et al [51] used a Fitbit to evaluate patients’ objectively measured PA.

#### Steps Per Day

Researchers in 11 studies assessed steps per day using an ActiGraph accelerometer [44,46,47,50,55,57,64,67,68,72,81]. Anderson et al [75] used a SenseWear PA monitor to assess steps per day, while Ferrante et al [51] and Walsh et al [76] used Fitbit devices. In addition, a pedometer was used to evaluate steps per day by Sajid et al [65] and Frensham et al [79].

#### Sedentary Behavior

##### Overview

Researchers in 13 studies used ActiGraph accelerometers to assess sedentary behavior [46,50,54,58,59,64,67,71,81,82].

##### Subjectively Measured Physical Activity

Six scales were used in 17 studies to assess cancer survivors’ subjectively measured PA: the International Physical Activity Questionnaire Short Form [75], the Community Healthy Activities Model Program for Seniors [49,57,62,77], the International Physical Activity Questionnaire [66,84,87], the Short Questionnaire to Assess Health-Enhancing Physical Activity [85], the Godin Leisure-Time Exercise Questionnaire [67,76,86], and the Seven-Day Physical Activity Recall [43,56,61-63].

##### Quality of Life

Nine scales were used to assess the QoL of cancer survivors in 22 studies: the Patient-Reported Outcome Measurement Information System [50], the 36-item Short Form Health Survey—Physical Component [46,61,73,77,79], the Quality of Life in Adult Cancer Survivors [51], the EORTC QLG Core Questionnaire-30 [56,60,66,85,87], the Functional Assessment of Cancer Therapy—General [48,88], the Functional Assessment of Cancer Therapy—Breast [44,58,74], the Functional Assessment of Cancer Therapy—Colorectal [62,86], the RAND-36 Measure of Health-Related Quality of Life [76], and the Pediatric Quality of Life Inventory [53,59].

### Feasibility

Researchers in 12 studies reported feasibility, which was assessed by retention rate (n=8), wearing time (n=2), whether steps per day improved or not (n=1), and adherence to interventions (n=1) [39-41,44,45,48,49,59,62,63,67,69,77,78].

### Risk of Bias

**Figure 2.**
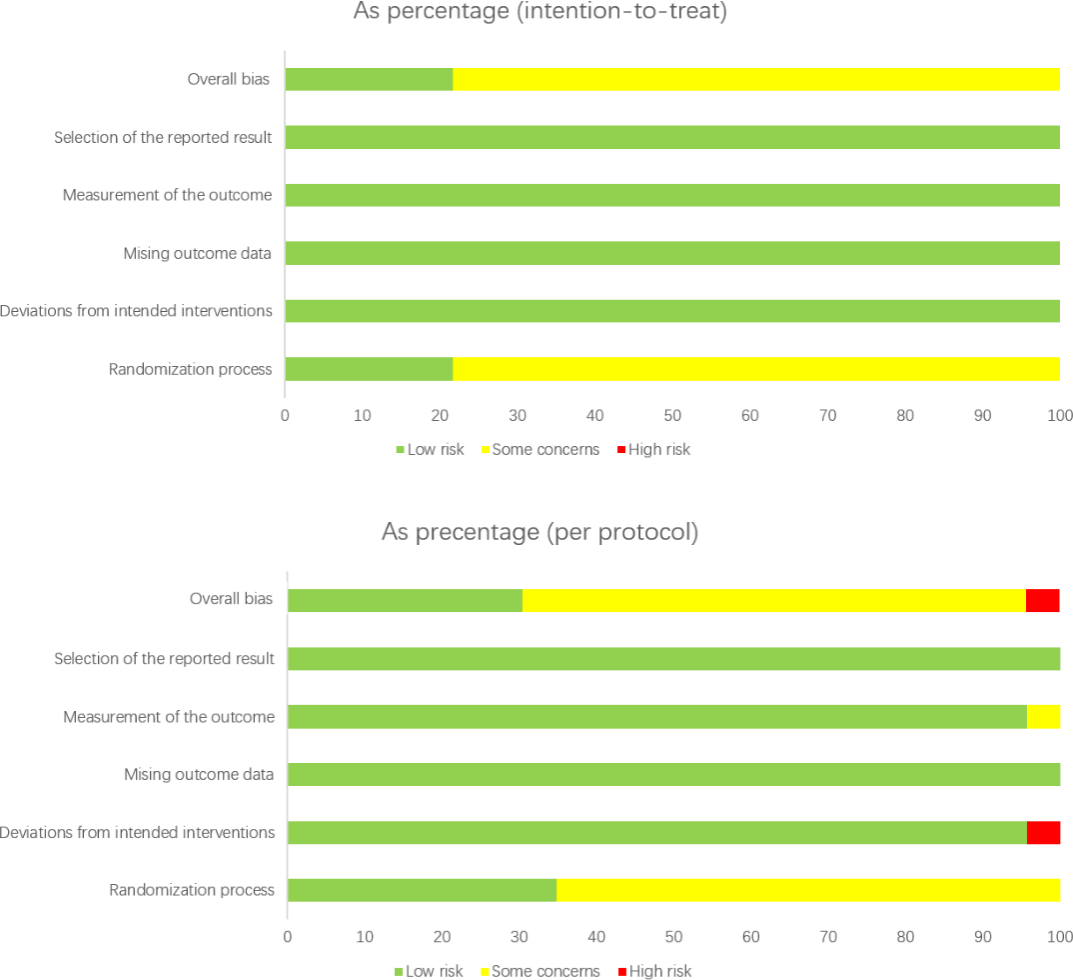
Results of the assessments of the risk of bias.

Utilizing the revised Cochrane risk-of-bias tool, the 24 studies that utilized intention-to-treat analysis within the inclusion criteria were classified as follows: 6 (25%) studies [44,51,55,77,78,84] were deemed to have a low risk of bias, while 18 (75%) studies [43,46,48,54,57,59,61,62,67,70,71,74,76,80,82,85,86,88] were identified as having some concerns regarding bias. Furthermore, among the studies that used per-protocol analysis (N=22), 6 (27%) studies [47,49,64,75,79,81] were assessed as having a low risk of bias, whereas 15 (68%) studies [50,52,53,56,58,60,63,65,66,68,69,72,73,83,87] were classified as having some concerns, and 1 (5%) study [45] was classified as having a high risk of bias. Concerns regarding risk of bias emerged due to the randomization process (33/47 studies) [43,46,48,50,52-54,56-63,65-74,76,80,82,83,85-88] and the measurement of the outcome (1 of 47 studies) [52]. A high risk of bias was associated with deviation from the intended interventions (1 of 47 studies) [45]. The assessments of risk of bias are comprehensively presented in Figure 2. In addition, the results of the Egger test revealed no evidence of small study effects (objectively measured MVPA: *P*=.26; subjectively reported PA: *P*=.09; steps per day: *P*=.12; sedentary behavior: *P*=.15; BMI: *P*=.13; QoL: *P*=.24; Multimedia Appendix 3).

### Meta-Analysis Results

The summary of all outcomes included in this meta-analysis is detailed in Multimedia Appendix 3.

#### Primary Outcome: Objectively Measured MVPA

##### Total Effects of WEDS-Supported PA Programs 

**Figure 3.**
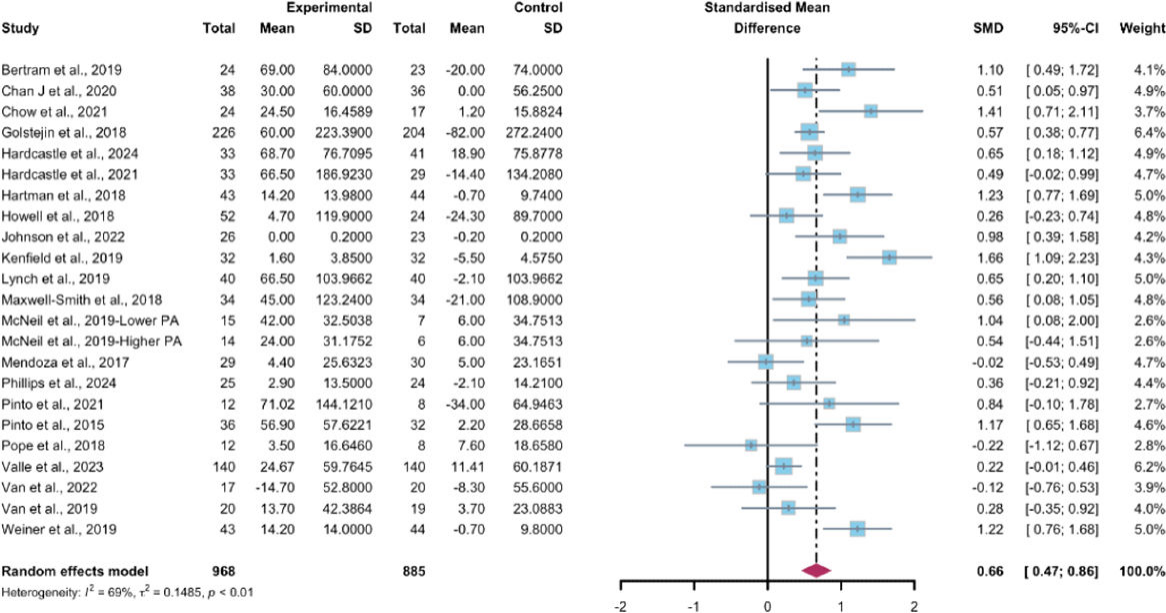
Total effects on objectively measured moderate-to-vigorous-intensity physical activity [43,46,48,49,51-54,57,58,62,63,66-68,70,71,78-81,84]. SMD: standardized mean difference.

Investigators from 23 studies, encompassing a total of 1853 participants, quantified the influence of WEDS-supported PA programs on the objectively reported MVPA among cancer survivors. The random-effects model used for pooling the data yielded a significant improvement in the intervention groups (SMD 0.66, 95% CI 0.47-0.86, *P*<.001, *I*^2^=69%; Figure 3). Additionally, the meta-analysis results remained stable after the omission of individual studies (Multimedia Appendix 4).

##### Subgroup Analysis

**Figure 4.**
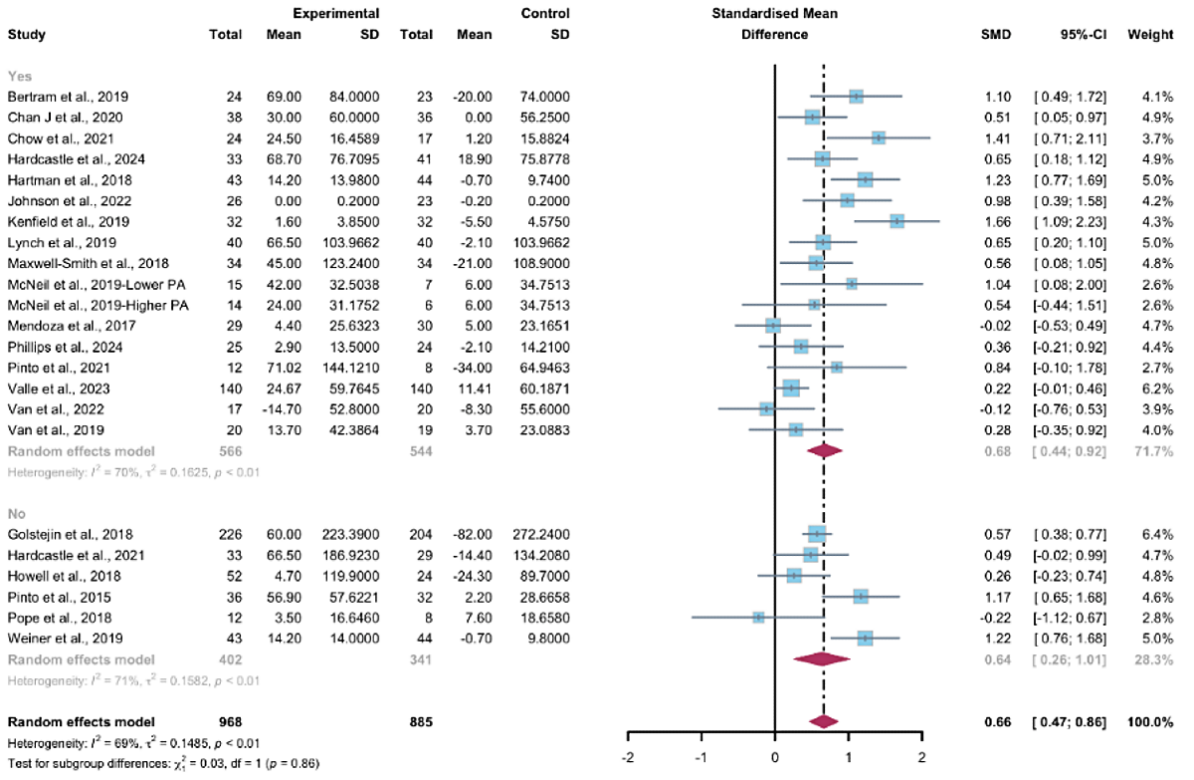
Subgroup analysis on objectively measured moderate-to-vigorous-intensity physical activity, grouped by the use of multipartnering tools [43,46,48,49,51-54,57,58,62,63,66-68,70,71,78-81,84]. SMD: standardized mean difference.

Studies grouped by the use of multipartnering tools suggested that WEDS-supported PA programs, whether with both multipartnering tools (SMD 0.68, 95% CI 0.44-0.92, *P*<.001, *I*^2^=70%) or without (SMD 0.63, 95% CI 0.26-1.01, *P*<.001, *I*^2^=71%), showed significant improvements in objectively measured MVPA (Figure 4).

**Figure 5.**
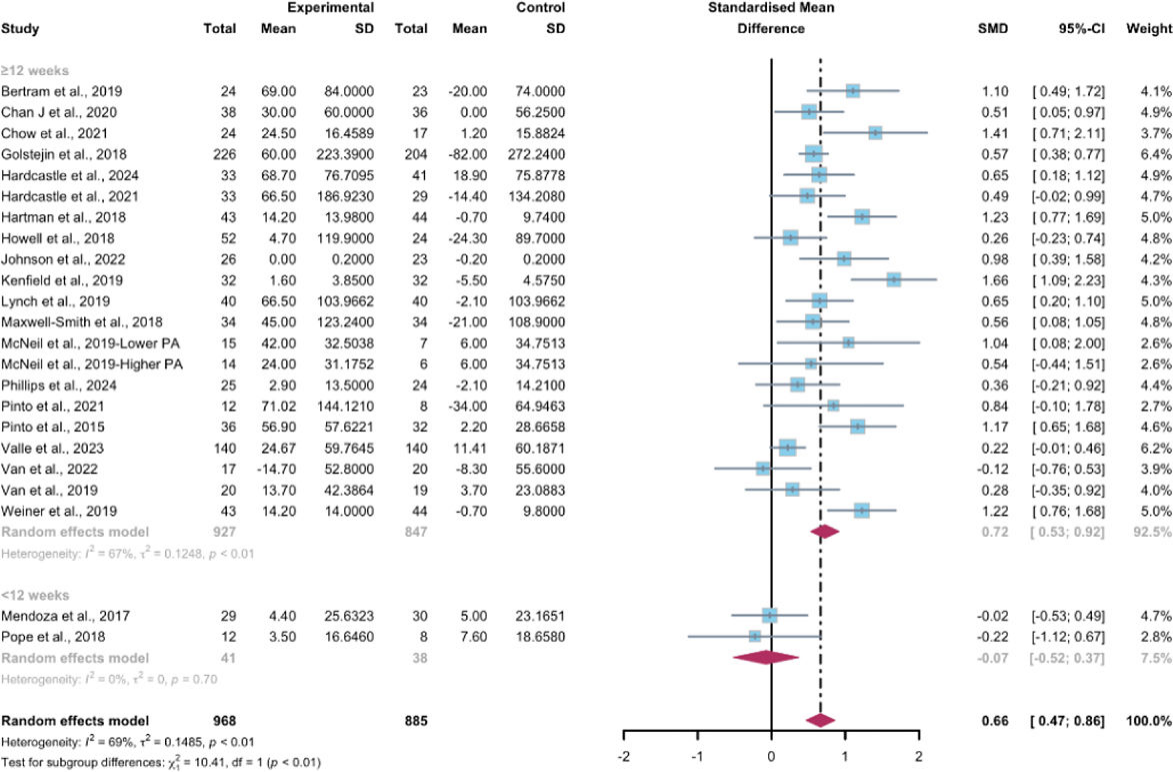
Subgroup analysis on objectively measured moderate-to-vigorous-intensity physical activity, grouped by intervention duration [43,46,48,49,51-54,57,58,62,63,66-68,70,71,78-81,84]. SMD: standardized mean difference.

**Figure 6.**
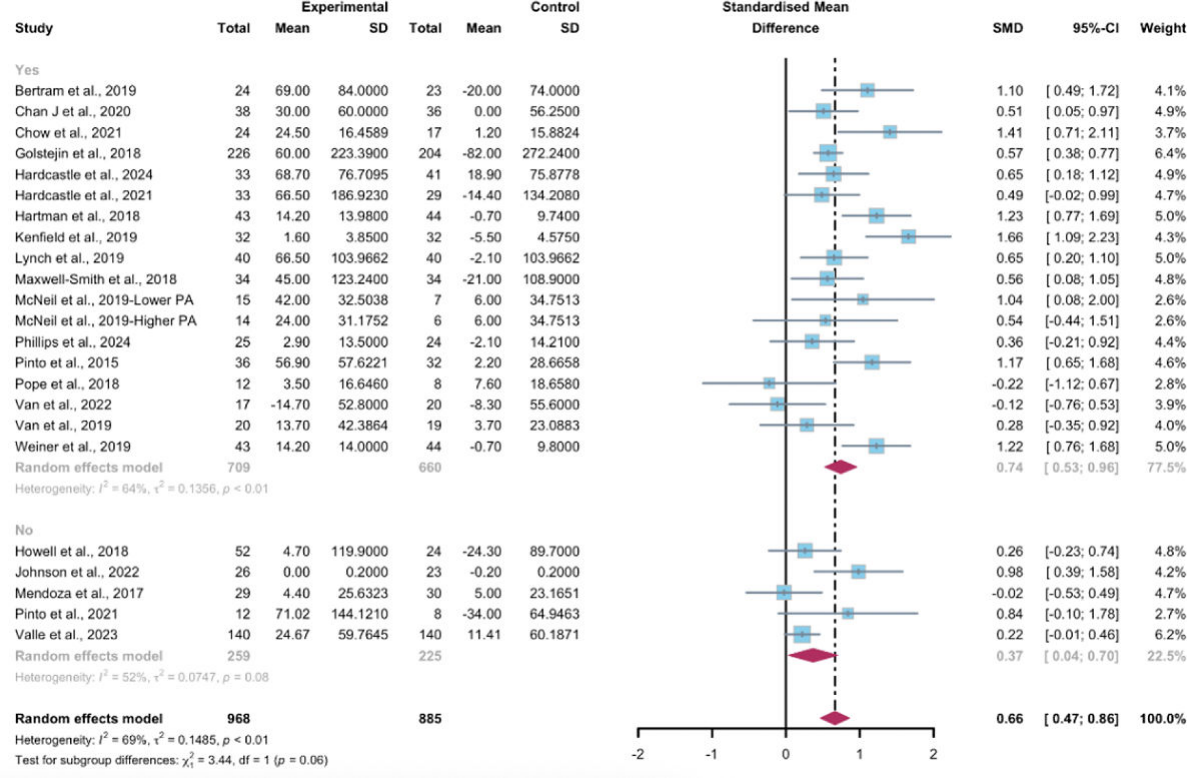
Subgroup analysis on objectively measured moderate-to-vigorous-intensity physical activity, grouped by whether the intervention was designed for a specific cancer type [43,46,48,49,51-54,57,58,62,63,66-68,70,71,78-81,84]. SMD: standardized mean difference.

Upon categorizing the studies based on the duration of the intervention, the pooled results indicated that WEDS-supported PA programs with long-term durations (≥12 weeks) were effective in increasing objectively measured MVPA (SMD 0.72, 95% CI 0.53-0.92, *P*<.001, *I*^2^=67%; Figure 5). When grouped by whether the intervention was designed for a specific cancer type, both the “yes” group (SMD 0.74, 95% CI 0.52-0.96, *P*<.001, *I*^2^=64%) and the “no” group (SMD 0.37, 95% CI 0.04-0.7, *P*=.02, *I*^2^=52%) showed significant differences (Figure 6). Heterogeneity in these 2 subgroups showed a modest to notable decrease (Multimedia Appendix 2). Duration and whether the intervention was designed for patients with a specific cancer type may be sources of heterogeneity.

### Secondary Outcomes: Subjectively Reported PA

#### Total Effects of WEDS-Supported PA Programs

**Figure 7.**
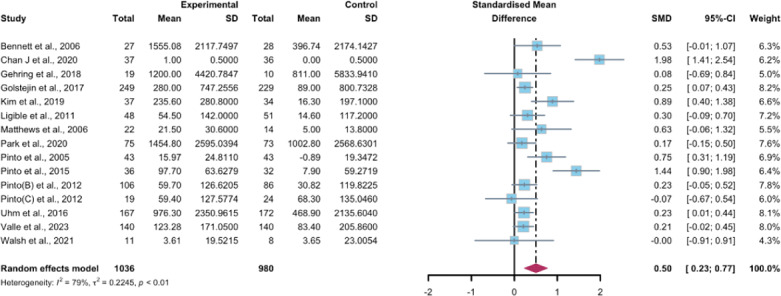
Total effects on subjectively reported physical activity [42,48,55,56,60-62,65,66,75,76,83-86]. SMD: standardized mean difference.

Data gathered from 15 studies, involving a total of 2016 participants, were used to assess the efficacy of WEDS-supported PA programs in increasing subjectively reported PA. The results of the random-effects model suggested a significant improvement in the experimental groups (SMD 0.5, 95% CI 0.23-0.77, *P*<.001, *I*^2^=79%; Figure 7). Furthermore, utilizing the one-study-out approach for sensitivity analysis, the findings remained stable (Multimedia Appendix 4).

#### Subgroup Analysis

**Figure 8.**
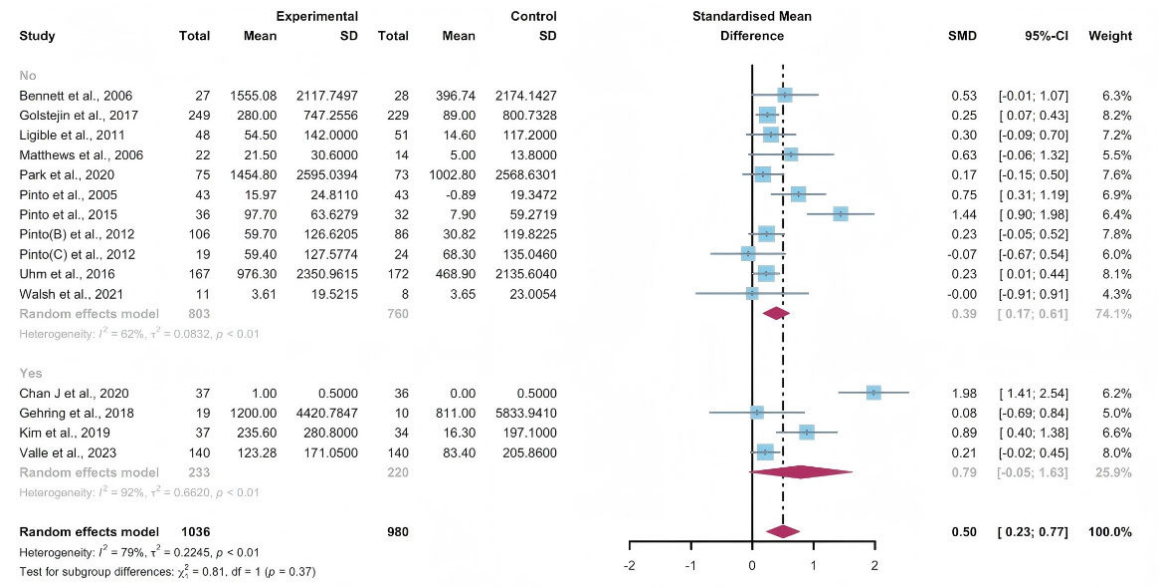
Subgroup analysis on subjectively reported physical activity, grouped by the use of multipartnering tools [42,48,55,56,60-62,65,66,75,76,83-86]. SMD: standardized mean difference.

In the pooled analysis based on the use of multipartnering tools, the results in the subgroup without multipartnering tools (SMD 0.39, 95% CI 0.17-0.61, *P*<.001, *I*^2^=62%) showed a significant improvement in subjectively reported PA (Figure 8).

**Figure 9.**
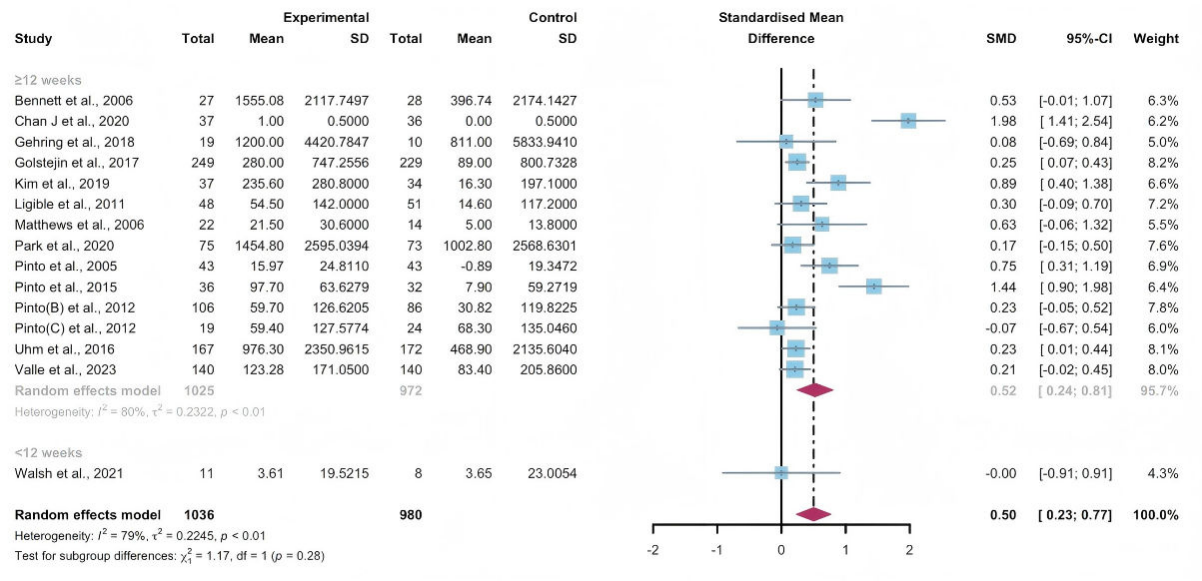
Subgroup analysis on subjectively reported physical activity, grouped by intervention duration [42,48,55,56,60-62,65,66,75,76,83-86]. SMD: standardized mean difference.

**Figure 10.**
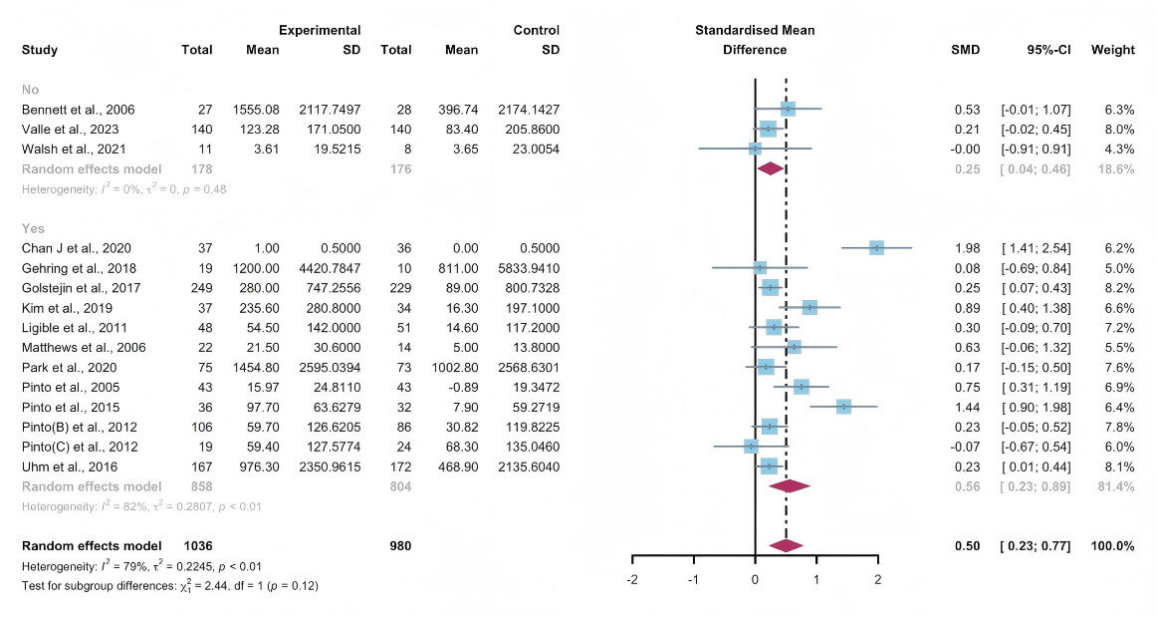
Subgroup analysis on subjectively reported physical activity, grouped by whether the intervention was designed for a specific cancer type [42,48,55,56,60-62,65,66,75,76,83-86]. SMD: standardized mean difference.

Upon categorizing the studies based on the duration of intervention, subjectively reported PA significantly increased in the long-term intervention groups (no less than 12 weeks; SMD 0.52, 95% CI 0.24-0.81, *P*<.001, *I*^2^=80%; Figure 9). When grouped by whether the intervention was designed for a specific cancer type, patients’ subjectively reported PA improved in both the “yes” group (SMD 0.56, 95% CI 0.23-0.89, *P*<.001, *I*^2^=82%) and the “no” group (SMD 0.25, 95% CI 0.04-0.06, *P*=.02, *I*^2^=0%; Figure 10). The use of multipartnering tools and whether interventions were designed for patients with specific cancer types may be sources of heterogeneity (Multimedia Appendix 2).

### Secondary Outcomes: Steps Per Day

#### Total Effects of WEDS-Supported PA Programs

**Figure 11.**
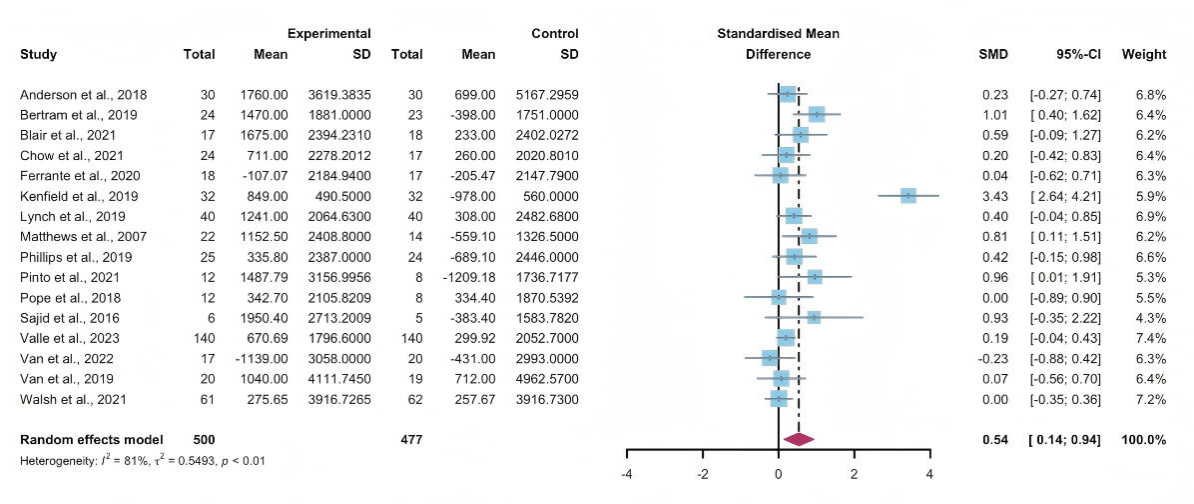
Total effects on steps per day [45,46,49,50,54,56,63,64,66-68,71,74,75,78,80]. SMD: standardized mean difference.

Fifteen studies assessed the impact of WEDS-supported PA programs on the steps per day of patients with cancer [44,46,47,50,55,57,64,65,67,68,72,75,76,79,81]. The random-effects model revealed a significant difference between the intervention and control groups (SMD 0.54, 95% CI 0.14-0.94, *P*=.002, *I*^2^=81%; Figure 11). Utilizing the one-study-out approach for sensitivity analysis, the pooled findings remained robust upon the sequential exclusion of individual studies (Multimedia Appendix 4).

#### Subgroup Analysis

**Figure 12.**
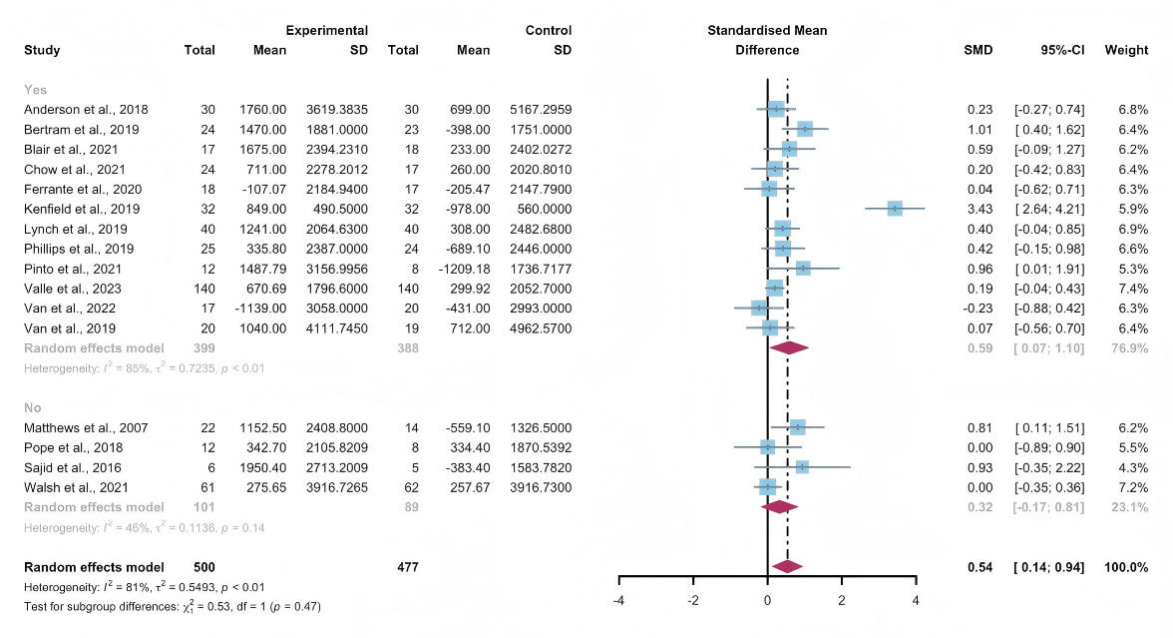
Subgroup analysis on steps per day, grouped by the use of multipartnering tools [45,46,49,50,54,56,63,64,66-68,71,74,75,78,80]. SMD: standardized mean difference.

In the pooled results of the partnering tools used in WEDS-supported PA programs, compared with the subgroup without multipartnering tools, the subgroup using multipartnering showed a significant difference in the number of steps per day (SMD 0.59, 95% CI 0.07-1.1, *P*=.006, *I*^2^=85%; Figure 12). Heterogeneity decreased in the subgroup without multipartnering tools (*I*^2^=46%).

**Figure 13.**
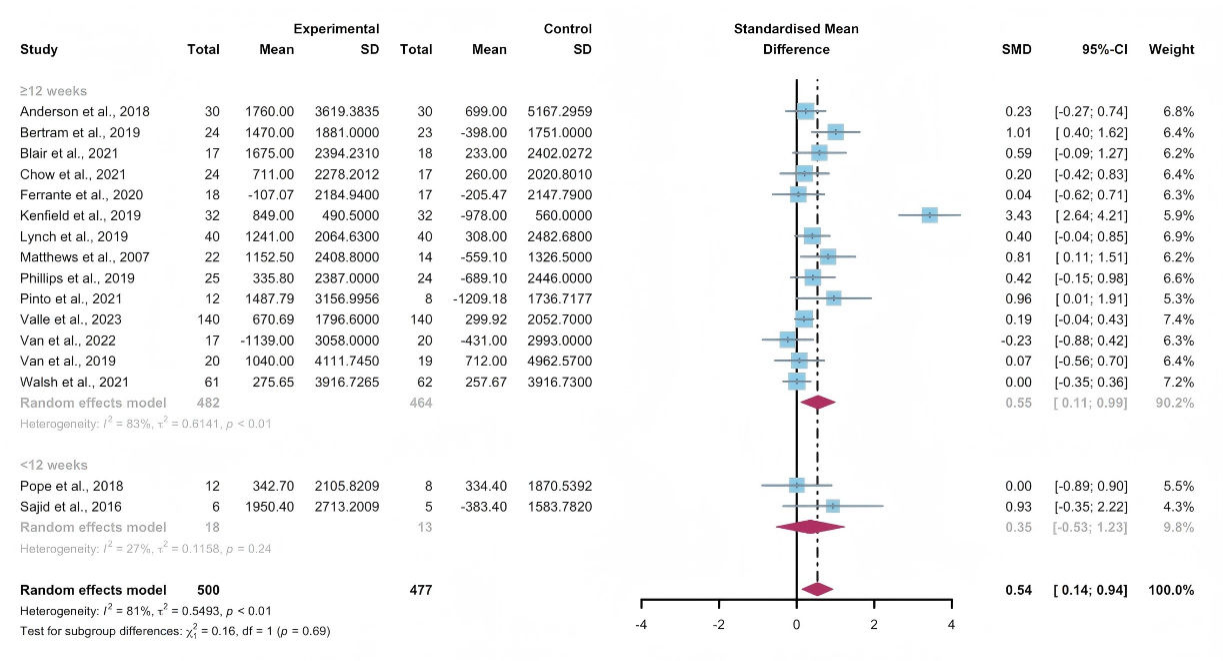
.Subgroup analysis on steps per day, grouped by intervention duration [45,46,49,50,54,56,63,64,66-68,71,74,75,78,80]. SMD: standardized mean difference.

The pooled findings from the subgroup analysis indicated a significant increase in steps per day when the duration of WEDS-supported PA programs was no less than 12 weeks (SMD 0.55, 95% CI 0.11-0.99, *P*=.003 *I*^2^=83%; Figure 13). Heterogeneity decreased in the group with a duration of less than 12 weeks (*I*^2^=27%).

**Figure 14.**
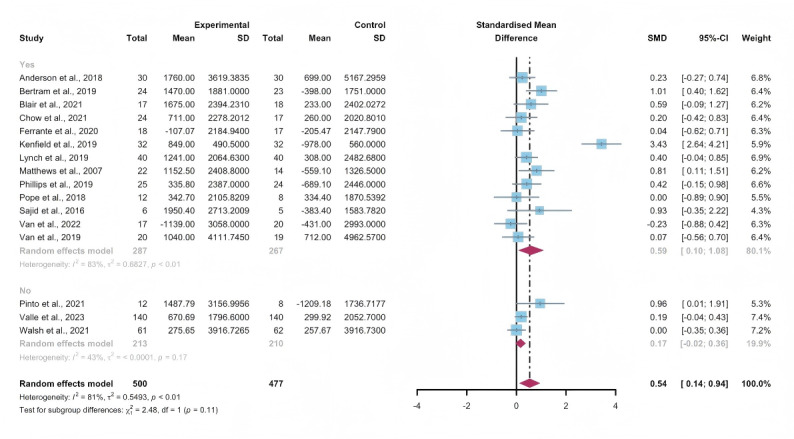
Subgroup analysis on steps per day, grouped by whether the intervention was designed for a specific cancer type [45,46,49,50,54,56,63,64,66-68,71,74,75,78,80]. SMD: standardized mean difference.

When grouped by whether the intervention was designed for a specific cancer type, patients’ steps per day improved in the “yes” group (SMD 0.59, 95% CI 0.1-1.08, *P*=.008, *I*^2^=83%; Figure 14). Heterogeneity decreased in the group without specific cancer types (*I*^2^=43%).

The use of multipartnering tools, intervention duration, and whether patients were allocated based on specific cancer types may be sources of heterogeneity.

### Secondary Outcomes: Sedentary Behavior

#### Total Effects of WEDS-Supported PA Programs

**Figure 15.**
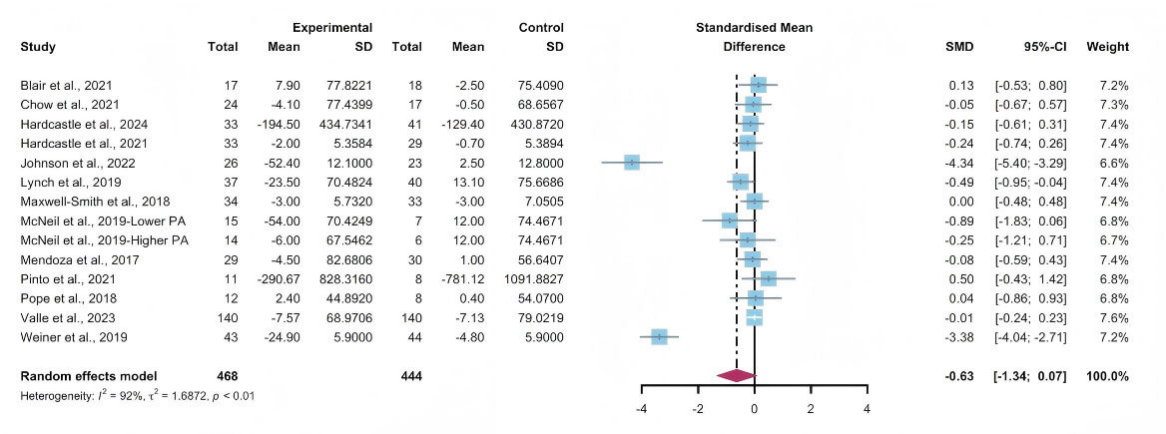
Total effects on sedentary behavior [43,45,49,53,57,58,63,66,70,71,79-81]. SMD: standardized mean difference.

Data gathered from 13 studies, involving a total of 912 participants, were used to assess the efficacy of WEDS-supported PA programs in decreasing sedentary behavior. The results of the random-effects model demonstrated that WEDS-supported PA programs did not significantly decrease cancer survivors’ sedentary behavior (SMD −0.63, 95% CI −1.34 to 0.07, *P*=.08, *I*^2^=92%; Figure 15). Utilizing the one-study-out approach for sensitivity analysis, the findings remained robust (Multimedia Appendix 4).

#### Subgroup Analysis

**Figure 16.**
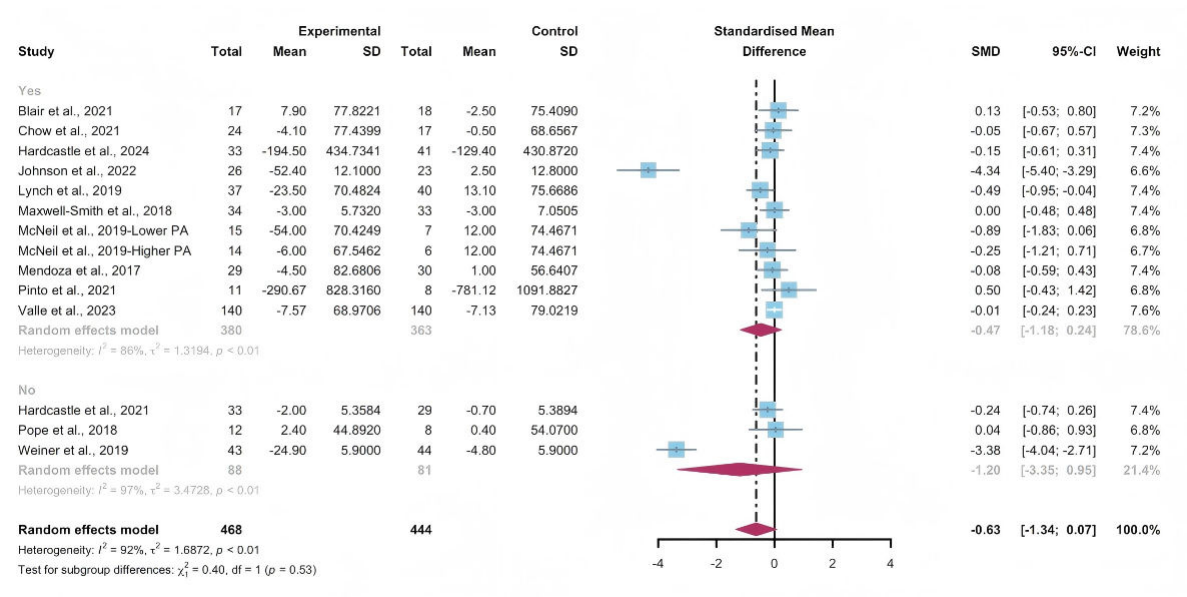
Subgroup analysis on sedentary behaviors, grouped by the use of multipartnering tools [43,45,49,53,57,58,63,66,70,71,79-81]. SMD: standardized mean difference.

**Figure 17.**
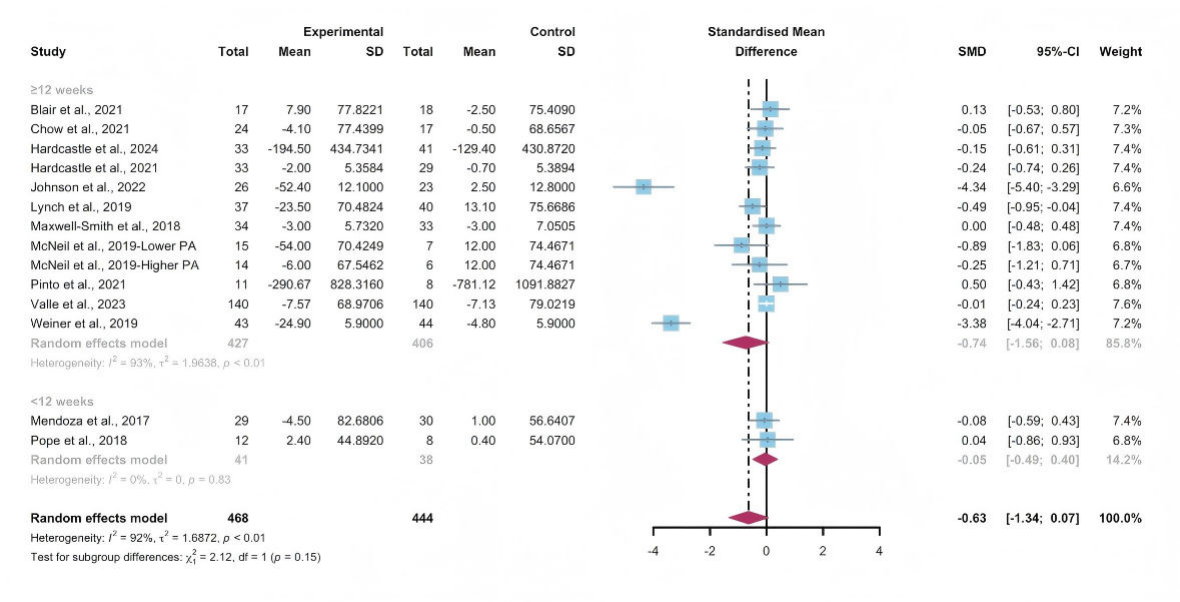
Subgroup analysis on sedentary behaviors, grouped by intervention duration [43,45,49,53,57,58,63,66,70,71,79-81]. SMD: standardized mean difference.

**Figure 18.**
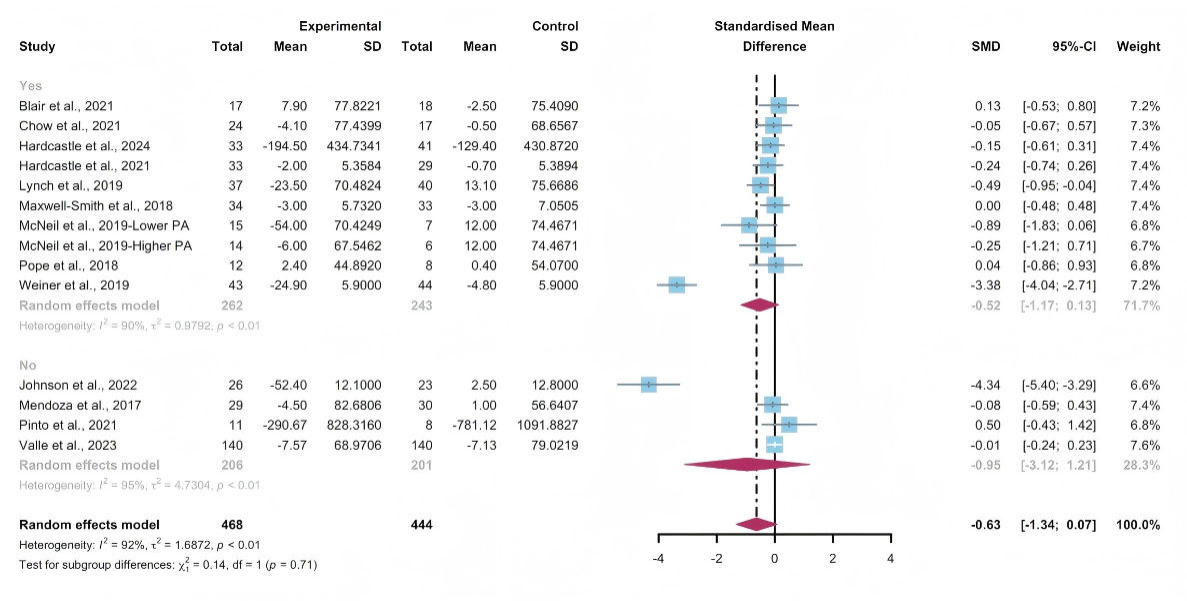
Subgroup analysis on sedentary behaviors, grouped by whether the intervention was designed for a specific cancer type [43,45,49,53,57,58,63,66,70,71,79-81]. SMD: standardized mean difference.

In the pooled results for the subgroups of WEDS-supported PA programs, usage of multipartnering tools, durations of interventions, and whether interventions were designed for specific cancer types, no significant differences were observed (Figures 16-18 and Multimedia Appendix 2).

Heterogeneity decreased in the group with a duration of less than 12 weeks (*I*^2^=0%).

### Secondary Outcomes: BMI

#### Total Effects of WEDS-Supported PA Programs on BMI 

**Figure 19.**
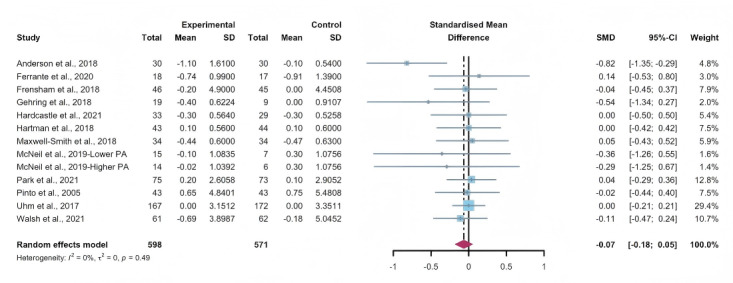
Total effects on BMI [42,50,51,57,65,74,75,77,79,81,83,86]. SMD: standardized mean difference.

Data gathered from 12 studies, involving a total of 1134 participants, were used to assess the efficacy of WEDS-supported PA programs in decreasing BMI. The results of the random-effects model demonstrated no significant difference between the experimental and control groups (SMD −0.07, 95% CI −0.18 to 0.05, *P*=.27, *I*^2^=0%; Figure 19). In addition, the pooled findings remained robust upon the sequential exclusion of individual studies (Multimedia Appendix 4).

#### Subgroup Analysis (BMI)

**Figure 20.**
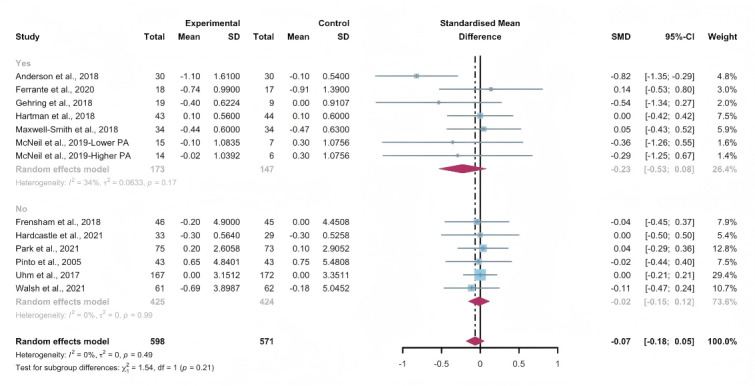
Subgroup analysis on BMI, grouped by the use of multipartnering tools [42,50,51,57,65,74,75,77,79,81,83,86]. SMD: standardized mean difference.

In the pooled analysis of subgroups within WEDS-supported PA programs, considering factors such as the use of multipartnering tools, the duration of interventions, and whether the interventions were tailored to specific cancer types, no significant differences were found (Figures20  21 and Multimedia Appendix 2).

**Figure 21.**
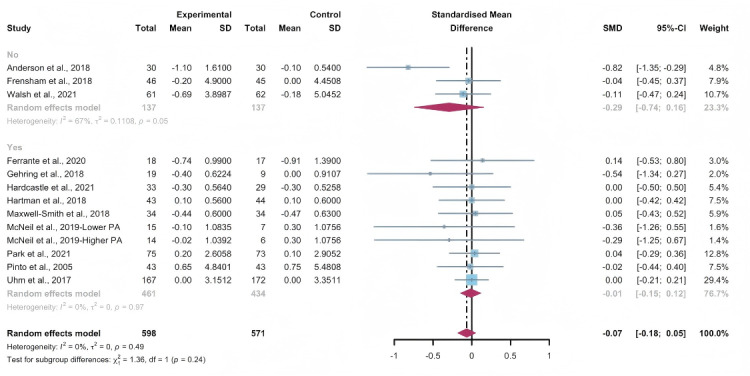
Subgroup analysis on BMI, grouped by whether the intervention was designed for a specific cancer type [42,50,51,57,65,74,75,77,79,81,83,86]. SMD: standardized mean difference.

### Secondary Outcomes: Quality of Life

#### Total Effects of WEDS-Supported PA Programs

**Figure 22.**
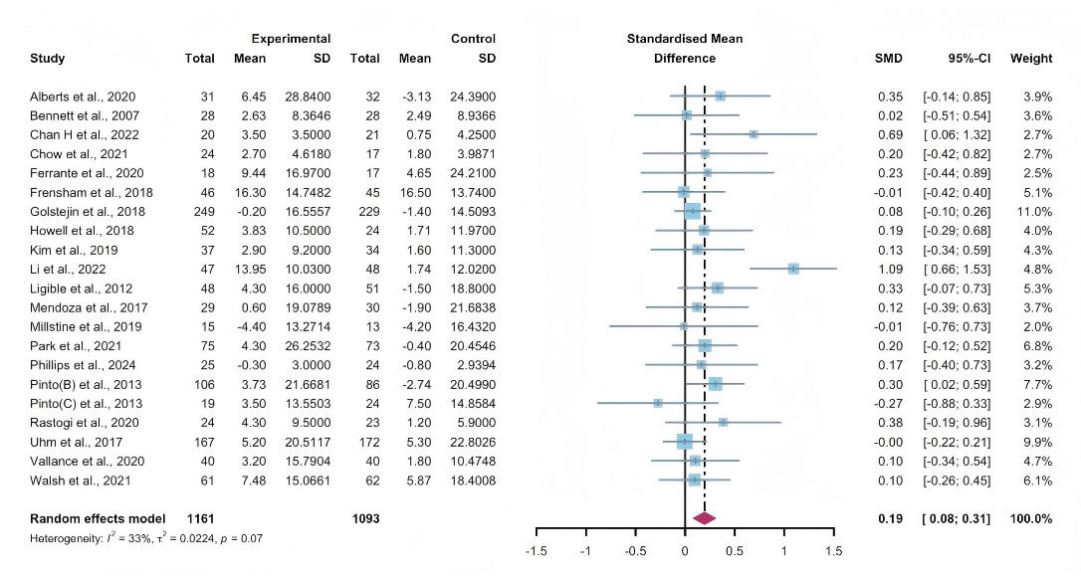
Total effects on quality of life. SMD: standardized mean difference. [36,44,47,49,50,52,55,58-61,65,72,73,75-78,84-86]. SMD: standardized mean difference.

Researchers from 21 studies assessed the effectiveness of WEDS-supported PA programs on the QoL of cancer survivors. The random-effects model revealed a significant difference between the experimental and control groups in the pooled results (SMD 0.19, 95% CI 0.08-0.31, *P*<.001, *I*^2^=33%; Figure 22). A sensitivity analysis was conducted using the one-study-out method, and the results remained robust.

#### Subgroup Analysis (QoL)

**Figure 23.**
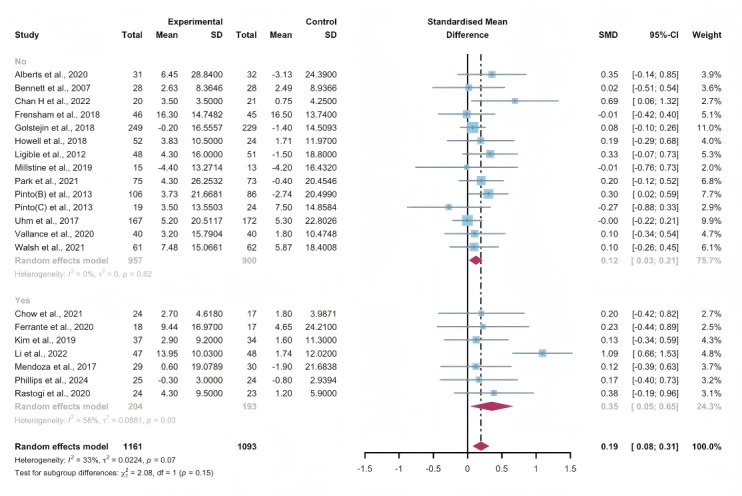
Subgroup analysis on quality of life, grouped by the use of multipartnering tools [36,44,47,49,50,52,55,58-61,65,72,73,75-78,84-86]. SMD: standardized mean difference.

In the pooled results based on the usage of multipartnering tools, both the subgroup using multipartnering tools (SMD 0.35, 95% CI 0.05-0.65, *P*<.001, *I*^2^=56%) and the subgroup not using them (SMD 0.12, 95% CI 0.03-0.21, *P*=.02, *I*^2^=0%) showed significant improvement in QoL (Figure 23). Notably, heterogeneity sharply decreased in the noncombination group (*I*^2^=0%).

**Figure 24.**
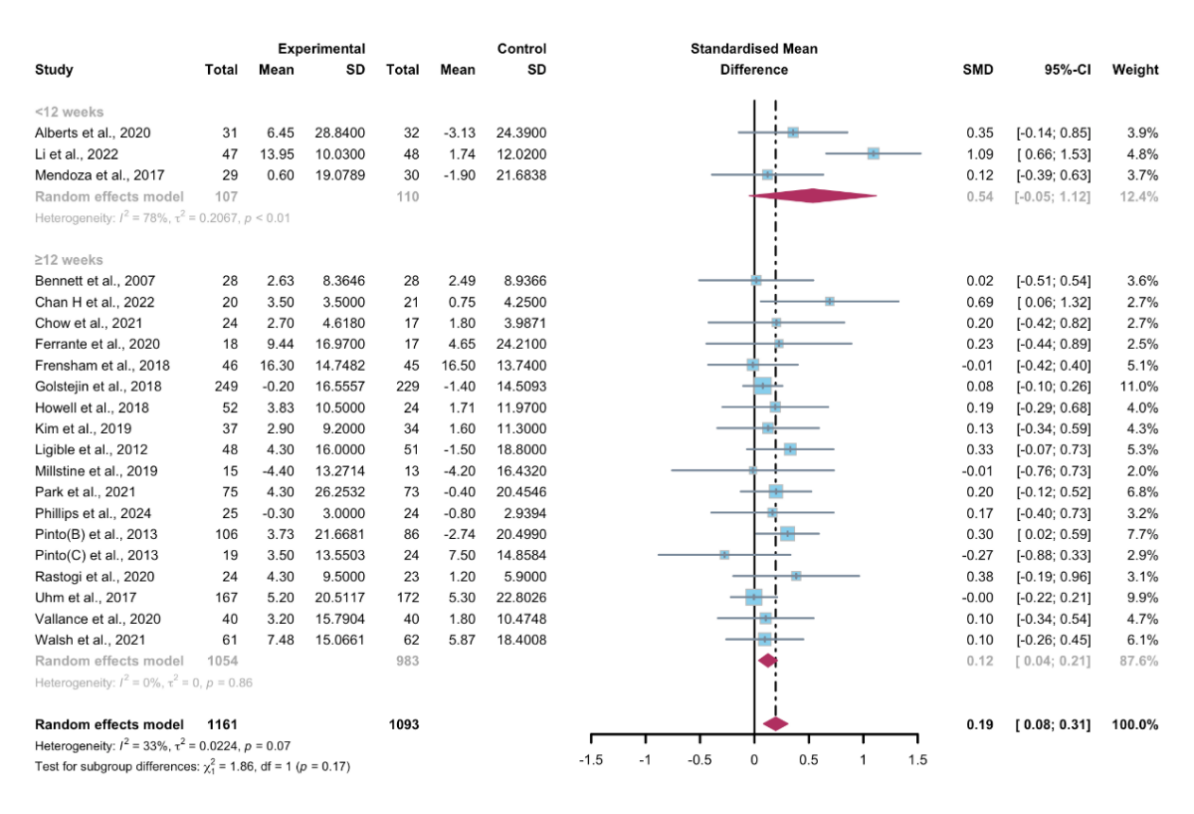
Subgroup analysis on quality of life, grouped by intervention duration [36,44,47,49,50,52,55,58-61,65,72,73,75-78,84-86]. SMD: standardized mean difference.

The pooled findings from the subgroup analysis indicated a significant increase in QoL when the duration of WEDS-supported PA programs was no less than 12 weeks, accompanied by a sharp decrease in heterogeneity (SMD 0.12, 95% CI 0.04-0.21, *P*<.001, *I*^2^=0%; Figure 24).

**Figure 25.**
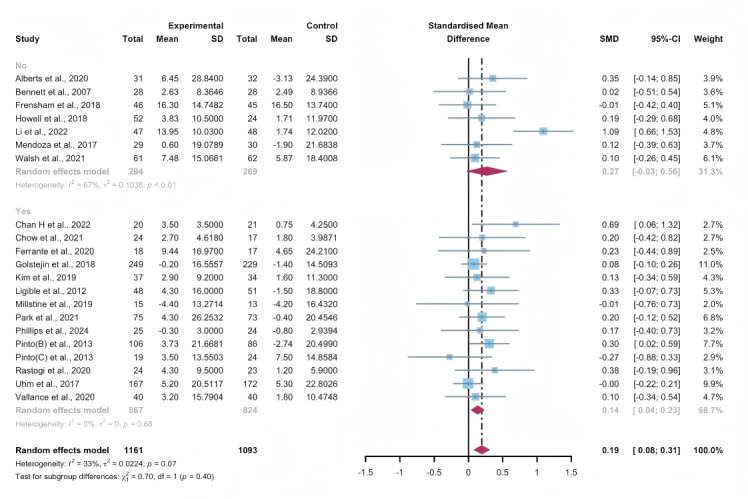
Subgroup analysis on quality of life, grouped by whether the intervention was designed for a specific cancer type [36,44,47,49,50,52,55,58-61,65,72,73,75-78,84-86]. SMD: standardized mean difference.

When grouped by whether the intervention was designed for a specific cancer type, patients’ QoL improved in the specific cancer type group (SMD 0.14, 95% CI 0.04-0.23, *P*=.006, *I*^2^=0%; Figure 25), with a sharp decrease in heterogeneity. Whether interventions were designed for a specific cancer type, whether multipartnering tools were used, and the duration of interventions might be potential sources of heterogeneity.

## Discussion

### Principal Findings

A total of 46 studies met the eligibility criteria for this meta-analysis. Compared with usual care or waitlists, WEDS-supported PA programs significantly improved cancer survivors’ objectively measured MVPA, subjectively measured PA, steps per day, and QoL, but showed no significant effect on reducing sedentary behavior or improving BMI. The SMD of total effects ranged from −0.07 to 0.66, which is consistent with previous studies [23,29], thereby confirming the effectiveness of WEDS-supported PA programs.

### Quality of Evidence and Methodology

Overall, the evidence quality and methodology were rated as moderate; 24 studies used the intention-to-treat analysis, while 22 utilized per-protocol analysis. Using the revised Cochrane Risk-of-Bias tool, it was determined that 12 of 46 (26%) studies were assessed as having a low risk of bias, 33 of 46 (72%) studies raised some concerns, and only 1 of 46 (2%) studies was deemed to have a high risk of bias. Specifically, of these 46 studies, 33 (72%) were flagged for potential bias related to the randomization process, and 1 (2%) raised concerns regarding bias in the measurement of outcomes. In detail, all studies were assessed as having either a low or unclear risk associated with the randomization process, primarily because some investigators failed to provide comprehensive details on the randomization techniques or adequately describe the allocation concealment methods. Consequently, the overall methodological quality was deemed moderate. These findings underscore the need for additional randomized controlled trials in the future, with a focus on more transparent reporting to enhance the robustness of research findings.

### PA-Related Outcomes

The results of this meta-analysis demonstrated that WEDS-supported PA programs significantly improved objectively measured MVPA, subjectively reported PA, steps per day, and QoL in cancer survivors, but had no significant effect on sedentary behavior or BMI. The findings related to objectively measured MVPA, subjectively reported PA, and steps per day are consistent with those of previous studies [23].

For cancer survivors, these interventions serve as tracking devices (continuously collecting current activity), feedback tools (providing immediate information on activity levels), and environmental cues (reminders to be active). Through these continuous influences, patients’ levels of PA increase [91]. In addition, the partnering tools used in these interventions enable cancer survivors to record, report, and contact HCPs anytime and anywhere they need, ensuring timely revision of exercise prescriptions and providing knowledge related to their disease and symptoms [78,80]. Moreover, the similar results between subjectively reported PA and objectively measured MVPA highlight the feasibility of WEDS-supported PA programs in improving PA among cancer survivors. On the contrary, we found that sedentary behavior did not improve significantly. This may be because patients may choose to ignore activity alarms while remaining sedentary [46]. More cognitive behavioral therapy is needed to enhance patients’ awareness and motivation to reduce sedentary behavior [92]. Regarding BMI, the lack of a significant difference might be due to patients gaining muscle mass, which can offset weight loss, resulting in no apparent change in BMI despite positive effects on fitness [93]. Furthermore, diet management has been shown to play a more significant role in weight loss programs than PA alone in many studies [94,95].

The results of the subgroup analysis on the usage of multipartnering tools revealed that objectively measured MVPA significantly improved regardless of whether multipartnering tools were used. This outcome may be because, at the beginning of the interventions, researchers set PA goals for participants and adjusted those goals based on their performance through the partnering tools. Whether or not multipartnering tools were used, the partnering tools could still serve as reminders for patients to complete more MVPA. Thus, the use of multipartnering tools may not have influenced objectively measured MVPA. By contrast, for subjectively reported PA, a significant difference was observed in the nonusage of multipartnering tools. When patients are assisted by multipartnering tools to remind them to improve PA, they may lack initiative, and their self-efficacy regarding PA may decrease, along with their perception of subjectively reported PA [96]. For steps per day, the multipartnering tools groups showed significant differences. This may be because various partnering tools play a more comprehensive role, such as providing real-time conversations through telephone calls and offering relevant knowledge via apps or websites. Through this type of multimedia stimulation, participants can receive more comprehensive reminders and encouragement to remain physically active.

Moreover, we observed significant improvements in objectively measured MVPA, subjectively reported PA, steps per day, and QoL among participants who received long-term (no less than 12 weeks) interventions, which is consistent with previous similar findings [36]. Research has shown that longer-term interventions are conducive to forming healthier lifestyles and developing lasting habits. Therefore, the patients can derive enjoyment from PA and are more willing to complete additional PA programs [97,98]. Similar to the overall effects, the results showed no significant improvements in sedentary behavior or BMI in the subgroups categorized by duration.

When studies were grouped by whether the intervention was designed for a specific cancer type, the results showed that both steps per day and QoL significantly improved in patients who received interventions tailored to their specific cancer type. This may be because tailored programs could better meet patients’ specific needs, such as providing information related to their cancer type and offering PA programs suited to their condition. By contrast, regardless of whether the interventions were tailored or not, the effectiveness of WEDS-supported PA programs in improving objectively measured MVPA, subjectively reported PA, sedentary behavior, and BMI did not change.

In brief, WEDS is promising for improving MVPA, subjectively reported PA, steps per day, and QoL. Long-term interventions (≥12 weeks) are effective in improving PA-related outcomes, except for sedentary behavior, and the use of multipartnering tools should depend on the patients’ preferences and habits. Attention should also be given to the proper use of multipartnering tools, the optimal duration, and whether the intervention is tailored to specific cancer types when developing new WEDS-supported interventions. Further studies are needed to explore the most effective intervention characteristics for improving patients’ sedentary behavior and BMI. This approach will facilitate the development of more effective WEDS-supported interventions.

### Quality of Life

This meta-analysis demonstrated that, compared with usual care or waitlists, WEDS-supported PA programs have a significant effect on the QoL of cancer survivors, which is consistent with the findings of previous studies [23]. The QoL assessed in the included studies was health-related QoL, which encompasses not only basic physical functioning but also patient participation in activities such as work and entertainment [99]. WEDS-supported PA programs significantly improved cancer survivors’ inactive lifestyles, enhanced their self-efficacy and feelings of self-worth, increased their satisfaction with life, and indirectly influenced their QoL [100]. Moreover, appropriate social relationships, cancer and self-care education, and psychological support provided through partnering tools could further help improve cancer survivors’ QoL [101,102].

In the subgroup analysis, regarding the use of multipartnering tools, QoL was significantly improved in both the usage and nonusage groups. This may be because interventions in both groups provided reminders to patients, which significantly enhanced participants’ PA levels and indirectly reduced their symptom burden, thereby improving QoL. When grouped by intervention duration, QoL was significantly improved in the long-term subgroups. Longer intervention durations enable patients to develop sustained habits of positive PA. Additionally, patients may have more opportunities to access diverse forms of support over the long term, which provides greater encouragement for engaging in PA and fosters the adoption of self-management strategies, thereby improving their QoL [103]. Furthermore, we observed a significant improvement in QoL among patients who received interventions designed for a specific cancer type. Researchers could tailor specific programs, such as PA regimens and psychological support from HCPs, according to the characteristics of each patient’s cancer.

In essence, WEDS-supported PA programs enable cancer survivors to engage positively with WEDs and partnering tools, with the potential to reduce negative affective states and consequently enhance their QoL. Researchers should carefully consider the duration of intervention when designing WEDS-supported strategies. Additionally, further investigation is warranted to evaluate the effectiveness of partnering tools in addressing the specific needs of cancer survivors.

### Limitations

This study has several limitations. First, heterogeneity existed due to variations in the format of partnering tools, durations of intervention, and types of cancer. Second, the reporting of study results may have been influenced by commercial interests associated with PA improvements, posing a potential risk of publication bias. Additionally, a significant proportion of the research was conducted in Western countries, and responses to WEDS-supported PA programs may vary among participants from different regions [104]. Finally, despite conducting an exhaustive literature search, publication bias could not be completely eliminated. Therefore, the outcomes of this meta-analysis should be interpreted with caution, and more high-quality randomized controlled trials are needed in the future.

### Implications

In this study, we quantitatively integrated existing findings and found that WEDS-supported PA programs were effective in improving PA levels (both objectively and subjectively), daily steps, and QoL. The mechanisms through which WEDS-supported PA programs bring clinical benefits may include providing persistent reminders to encourage PA, offering convenient access to consultations with HCPs, collecting health-related data, recording electronic health records, and facilitating social groups for patients to communicate with others facing similar conditions [47,50]. Thus, HCPs can use WEDS as a supplementary tool to monitor patients’ physiological data, manage care, adjust exercise prescriptions, and provide timely feedback and disease-related information.

With increasing research focusing on WEDs and other forms of eHealth as interventions to promote PA among cancer survivors, WEDS has the potential to become a valuable tool for HCPs and a novel reminder and management resource for cancer survivors. It can automatically sync data, thereby reducing the self-monitoring burden associated with traditional web-based interventions [105]. Additionally, previous studies often failed to adequately consider the role of partnering tools, resulting in their underutilization and a missed opportunity to maximize the benefits for patients’ PA engagement. Furthermore, we observed that certain aspects of the intervention, such as the use of multipartnering tools, the duration of the intervention, and whether the intervention was tailored for specific cancer types, influenced its overall efficacy. This underscores the need for further standardization and more rigorous quantitative studies to refine the WEDS-supported intervention framework and to fully explore the potential benefits of WEDS-supported PA programs. Moreover, efforts should be made to enable data intercommunication between different commercial WEDs, thereby improving the feasibility and accessibility of these interventions.

### Conclusions

WEDS-supported PA programs offer a convenient and affordable method for assisting cancer survivors by serving as reminders and records of their PA. This meta-analysis of randomized controlled trials revealed that WEDS-supported PA programs significantly improved cancer survivors’ level of PA (both objectively and subjectively), steps per day, and QoL, but had no significant effect on reducing sedentary behavior or BMI. These results varied based on the use of multipartnering tools, intervention duration, and patients’ cancer type. Further standardization and promotion of WEDS-supported PA programs are warranted in the future.

## Supplementary material

10.2196/74347 Multimedia Appendix 1 Search strategies.

10.2196/74347 Multimedia Appendix 2 Characteristics of included studies.

10.2196/74347 Multimedia Appendix 3 Summary of all outcomes included in the meta-analysis.

10.2196/74347 Multimedia Appendix 4 Results of the sensitivity analysis.

10.2196/74347 Checklist 1 PRISMA (Preferred Reporting Items for Systematic Reviews and Meta-Analyses) checklist.

## Abbreviations

HCP: health care professional
MeSH: Medical Subject Headings
mHealth: mobile health
MVPA: moderate-to-vigorous-intensity physical activity
PA: physical activity
PICOS: Participants, Interventions, Comparisons, Outcomes, and Study Design
PRISMA: Preferred Reporting Items for Systematic Reviews and Meta-Analyses
QoL: quality of life
SMD: standard mean difference
WED: wearable electronic device
WEDS: wearable electronic device system
