# Supplementary material for: The Effectiveness of Wearable Electronic Device System–Supported Physical Activity Programs for Cancer Survivors: Meta-Analysis of Randomized Controlled Trials
Source: J Med Internet Res. 2025 Aug 14;27:e74347. doi: 10.2196/74347 (PMC12352708; doi:10.2196/74347)
Supplement: Multimedia Appendix 1 [file jmir-v27-e74347-s001.docx]

| **Database** | **Search Strategies** |
| --- | --- |
| PubMed  814 | #1 Search: “wearable electronic devices”[MeSH Terms]  #2 Search: “Neoplasms”[Mesh Terms]  #3 Search: “Carcinoma”[Mesh Terms]  #4 Search: ((((((((((((((((((((((((((((((((((((wearable technolog*[Title/Abstract])) OR (wearable sensor*[Title/Abstract])) OR (wearable monitor*[Title/Abstract])) OR (wireless device*[Title/Abstract])) OR (wireless monitor*[Title/Abstract])) OR (activity tracker*[Title/Abstract])) OR (activity monitor*[Title/Abstract])) OR (fitness tracker*[Title/Abstract])) OR (fitness monitor*[Title/Abstract])) OR (smartwatch*[Title/Abstract])) OR (fitbit*[Title/Abstract])) OR (apple watch*[Title/Abstract])) OR (Jawbone*[Title/Abstract])) OR (Pebble*[Title/Abstract])) OR (pedometer*[Title/Abstract])) OR (accelerometer*[Title/Abstract])) OR (Actigraph*[Title/Abstract])) OR (remote monitor*[Title/Abstract])) OR (ambulatory monitor*[Title/Abstract])) OR (outpatient monitor*[Title/Abstract])) OR (actiwatch*[Title/Abstract]))) OR (wrist sensor[Title/Abstract])) OR (smart wristband[Title/Abstract])) OR (smart ring[Title/Abstract])) OR (electronic sensor[Title/Abstract]) OR (smart glass[Title/Abstract])) OR (smart bracelet[Title/Abstract])) OR (smart helmet[Title/Abstract])) OR (wireless networks[Title/Abstract])) OR (Bluetooth technology[Title/Abstract])) OR (smart headband[Title/Abstract])) OR (Google glasses[Title/Abstract])) OR (Limb-worn devices[Title/Abstract])) OR (wearable clothing[Title/Abstract])) OR (electronic skin[Title/Abstract])) OR (head-worn devices[Title/Abstract])  #5 Search: ((((((((((((((((((((((((((((((neoplas*[Title/Abstract])) OR (oncolog*[Title/Abstract])) OR (cancer*[Title/Abstract])) OR (tumor*[Title/Abstract])) OR (tumour*[Title/Abstract])) OR (carcinom*[Title/Abstract])) OR (melanom*[Title/Abstract])) OR (lymphom*[Title/Abstract])) OR (leumemi*[Title/Abstract])) OR (malignan*[Title/Abstract])) OR (metasta*[Title/Abstract])) OR (carcinogen*[Title/Abstract])) OR (oncogen*[Title/Abstract])) OR (anticarcinogen*[Title/Abstract])) OR (sarcoma*[Title/Abstract])) OR (blastoma*[Title/Abstract])) OR (meningioma*[Title/Abstract])) OR (lymphangioma*[Title/Abstract])) OR (lymphangiomyoma*[Title/Abstract])) OR (lymphangiosarcoma*[Title/Abstract])) OR (hodgkin disease*[Title/Abstract])) OR (plasmacytoma*[Title/Abstract])) OR (carcinosarcoma*[Title/Abstract])) OR (hepatoblastoma*[Title/Abstract])) OR (mesenchymoma*[Title/Abstract])) OR (chordoma*[Title/Abstract])) OR (germinoma*[Title/Abstract])) OR (gonadoblastoma*[Title/Abstract])) OR (mesonephroma*[Title/Abstract])) OR (teratoma[Title/Abstract]) OR (teratocarcinoma*[Title/Abstract])  #6 Search:  telerehabilitation[MeSH Terms]  #7 Search: telemedicine[MeSH Terms]  #8 Search: internet-based intervention[MeSH Terms]  #9 Search: (((((((((((((((((((((((((((((((((internet[Title/Abstract]) OR (web[Title/Abstract])) OR (internet based[Title/Abstract])) OR (internet-based[Title/Abstract])) OR (web based[Title/Abstract])) OR (web-based[Title/Abstract])) OR (webcasts[Title/Abstract])) OR (online[Title/Abstract])) OR (social media[Title/Abstract])) OR (social networking[Title/Abstract])) OR (telecommunications[Title/Abstract])) OR (telecommunication[Title/Abstract])) OR (mobile technology[Title/Abstract])) OR (mobile device[Title/Abstract])) OR (mobile app[Title/Abstract])) OR (phone[Title/Abstract])) OR (mobile phone[Title/Abstract])) OR (smart phone[Title/Abstract])) OR (cell phone[Title/Abstract])) OR (celluar phone[Title/Abstract])) OR (blog[Title/Abstract])) OR (blogging[Title/Abstract])) OR (SMS[Title/Abstract])) OR (computer[Title/Abstract])) OR (tablet[Title/Abstract])) OR (tablet computer[Title/Abstract])) OR (video[Title/Abstract])) OR (video recording[Title/Abstract])) OR (video conference[Title/Abstract])) OR (video conferencing[Title/Abstract])) OR (video meeting[Title/Abstract])) OR (interactive video[Title/Abstract])) OR (TV meeting[Title/Abstract])) OR (webcast[Title/Abstract])  #10 Search: ((((((((((((((((((((((((ehealth[Title/Abstract]) OR (telemedicine[Title/Abstract])) OR (telecare[Title/Abstract])) OR (telehomecare[Title/Abstract])) OR (telerehabilitation[Title/Abstract])) OR (telehealth[Title/Abstract])) OR (mhealth[Title/Abstract])) OR (telemonitor[Title/Abstract])) OR (telemanagement[Title/Abstract])) OR (mobile health[Title/Abstract])) OR (mhealth[Title/Abstract])) OR (mcare[Title/Abstract])) OR (mobile communication[Title/Abstract])) OR (remote consult*[Title/Abstract])) OR (e-health[Title/Abstract])) OR (tele-medicine[Title/Abstract])) OR (tele-care[Title/Abstract])) OR (tele-homecare[Title/Abstract])) OR (tele-rehabilitation[Title/Abstract])) OR (tele-health[Title/Abstract])) OR (m-health[Title/Abstract])) OR (tele-monitor[Title/Abstract])) OR (tele-management[Title/Abstract])) OR (m-health[Title/Abstract])) OR (m-care[Title/Abstract])  #11 Search: #6 OR #7 OR #8 OR #9 OR #10  #12 Search: #2 OR #3 OR #5  #13 Search: #4 AND #11  #14 Search: #1 OR #13  #15 Search: #12 AND #14 |
| Web of Science  795 | #1 Search: ((((((((((((((((((((((((((((((TS=(neoplas*)) OR TS=(oncolog*)) OR TS=(cancer*)) OR TS=(tumor*)) OR TS=(tumour*)) OR TS=(carcinom*)) OR TS=(melanom*)) OR TS=(lymphom*)) OR TS=(leumemi*)) OR TS=(malignan*)) OR TS=(metasta*)) OR TS=(carcinogen*)) OR TS=(oncogen*)) OR TS=(anticarcinogen*)) OR TS=(sarcoma*)) OR TS=(blastoma*)) OR TS=(meningioma*)) OR TS=(lymphangioma*)) OR TS=(lymphangiomyoma*)) OR TS=(lymphangiosarcoma*)) OR TS=(hodgkin disease*)) OR TS=(plasmacytoma*)) OR TS=(carcinosarcoma*)) OR TS=(hepatoblastoma*)) OR TS=(mesenchymoma*)) OR TS=(chordoma*)) OR TS=(germinoma*)) OR TS=(gonadoblastoma*)) OR TS=(mesonephroma*)) OR TS=(teratoma)) OR TS=(teratocarcinoma*)  #2 Search: ((((((((((((((((((((((((((((((((((((TI=(wearable electronic devices)) OR TI=(wearable technolog*)) OR TI=(wearable sensor*)) OR TI=(wearable monitor*)) OR TI=(wireless device*)) OR TI=(wireless monitor*)) OR TI=(activity tracker*)) OR TI=(activity monitor*)) OR TI=(fitness tracker*)) OR TI=(fitness monitor*)) OR TI=(smartwatch*)) OR TI=(fitbit*)) OR TI=(apple watch*)) OR TI=(Jawbone*)) OR TI=(Pebble*)) OR TI=(pedometer*)) OR TI=(accelerometer*)) OR TI=(Actigraph*)) OR TI=(remote monitor*)) OR TI=(ambulatory monitor*)) OR TI=(outpatient monitor*)) OR TI=(actiwatch*)) OR TI=(wrist sensor)) OR TI=(smart wristband)) OR TI=(smart ring)) OR TI=(electronic sensor)) OR TI=(smart glass)) OR TI=(smart braceleTI)) OR TI=(smart helmeTI)) OR TI=(wireless networks)) OR TI=(Bluetooth technology)) OR TI=(smart headband)) OR TI=(Google glasses)) OR TI=(Limb-worn devices)) OR TI=(wearable clothing)) OR TI=(electronic skin)) OR TI=(head-worn devices)  #3 Search: ((((((((((((((((((((((((TS=(ehealth)) OR TS=(telemedicine)) OR TS=(telecare)) OR TS=(telehomecare)) OR TS=(telerehabilitation)) OR TS=(telehealth)) OR TS=(mhealth)) OR TS=(telemonitor)) OR TS=(telemanagement)) OR TS=(mobile health)) OR TS=(mhealth)) OR TS=(mcare)) OR TS=(mobile communication)) OR TS=(remote consult*)) OR TS=(e-health)) OR TS=(tele-medicine)) OR TS=(tele-care)) OR TS=(tele-homecare)) OR TS=(tele-rehabilitation)) OR TS=(tele-health)) OR TS=(m-health)) OR TS=(tele-monitor)) OR TS=(tele-management)) OR TS=(m-health)) OR TS=(m-care)  #4 Search: (((((((((((((((((((((((((((((((((TS=(internet)) OR TS=(web)) OR TS=(internet based)) OR TS=(internet-based)) OR TS=(web based)) OR TS=(web-based)) OR TS=(webcasts)) OR TS=(online)) OR TS=(social media)) OR TS=(social networking)) OR TS=(telecommunications)) OR TS=(telecommunication)) OR TS=(mobile technology)) OR TS=(mobile device)) OR TS=(mobile app)) OR TS=(phone)) OR TS=(mobile phone)) OR TS=(smart phone)) OR TS=(cell phone)) OR TS=(celluar phone)) OR TS=(blog)) OR TS=(blogging)) OR TS=(SMS)) OR TS=(computer)) OR TS=(tablet)) OR TS=(tablet computer)) OR TS=(video)) OR TS=(video recording)) OR TS=(video conference)) OR TS=(video conferencing)) OR TS=(video meeting)) OR TS=(interactive video)) OR TS=(TV meeting)) OR TS=(webcast)  #5 Search: #3 OR #4  #6 Search: #2 AND #5  #7 Search: #1 AND #6 |
| Ovid Platform-Embase and MEDLINE  1814 | #1 Search: (neoplas* or oncolog* or cancer* or tumor* or tumour* or carcinom* or melanom* or lymphom* or leumemi* or malignan* or metasta* or carcinogen* or oncogen* or anticarcinogen* or sarcoma* or blastoma* or meningioma* or lymphangioma* or lymphangiomyoma* or lymphangiosarcoma* or hodgkin disease* or plasmacytoma* or carcinosarcoma or hepatoblastoma* or mesenchymoma* or chordoma* or germinoma* or gonadoblastoma* or mesonephroma* or teratoma or teratocarcinoma*).mp. [mp=title, abstract, heading word, drug trade name, original title, device manufacturer, drug manufacturer, device trade name, keyword heading word, floating subheading word, candidate term word]  #2 Search: (wearable electronic devices or wearable technolog* or wearable sensor* or wearable monitor* or wireless device or wireless monitor* or activity tracker* or activity monitor* or fitness tracker* or fitness monitor* or smartwatch* or fitbit* or apple watch* or Jawbone* or Pebble* or pedometer* or accelerometer* or Actigraph* or remote monitor* or ambulatory monitor* or outpatient monitor* or actiwatch* or wrist sensor or smart wristband or smart ring or electronic sensor or smart glass or smart braceleTI or smart helmeTI or wireless networks or Bluetooth technology or smart headband or Google glasses or Limb-worn devices or wearable clothing or electronic skin or head-worn devices).mp. [mp=title, abstract, heading word, drug trade name, original title, device manufacturer, drug manufacturer, device trade name, keyword heading word, floating subheading word, candidate term word]  #3 Search: (internet or web or internet based or internet-based or web based or web-based or webcasts or online or social media or social networking or telecommunications or telecommunication or mobile technology or mobile device or mobile app or phone or mobile phone or smart phone or cell phone or celluar phone or blog or blogging or SMS or computer or tablet or tablet computer or video or video recording or video conference or video conferencing or video meeting or interactive video or TV meeting or webcast or ehealth or telemedicine or telecare or telehomecare or telerehabilitation or telehealth or mhealth or telemonitor or telemanagement or mobile health or mhealth or mcare or mobile communication or remote consult* or e-health or tele-medicine or tele-care or tele-homecare or tele-rehabilitation or tele-health or m-health or tele-monitor or tele-management or m-health or m-care).mp. [mp=title, abstract, heading word, drug trade name, original title, device manufacturer, drug manufacturer, device trade name, keyword heading word, floating subheading word, candidate term word]  #4 Search: #2 AND #3  #5 Search: #1 AND #4 |
| CENTRAL  1131 | #1 Search: (wearable technolog*):ti,ab,kw OR (wearable sens*):ti,ab,kw OR (wearable monit*):ti,ab,kw OR (wireless device*):ti,ab,kw OR (wireless monit*):ti,ab,kw OR (activity tracker*):ti,ab,kw OR (activity monit*):ti,ab,kw OR (fitness tracker*):ti,ab,kw OR (fitness monit*):ti,ab,kw OR (smartwatch*):ti,ab,kw OR (fitbit*):ti,ab,kw OR (apple watch*):ti,ab,kw OR (Jawbone*):ti,ab,kw OR (Pebble*):ti,ab,kw OR (pedometer*):ti,ab,kw OR (accelerometer*):ti,ab,kw OR (Actigraph*):ti,ab,kw OR (remote monit*):ti,ab,kw OR (ambulaty monit*):ti,ab,kw OR (outpatient monit*):ti,ab,kw OR (actiwatch*):ti,ab,kw OR (wrist sens):ti,ab,kw OR (smart wristband):ti,ab,kw OR (smart ring):ti,ab,kw OR (electronic sens):ti,ab,kw OR (smart glass):ti,ab,kw OR (smart braceleTI):ti,ab,kw OR (smart helmeTI):ti,ab,kw OR (wireless netwks):ti,ab,kw OR (Bluetooth technology):ti,ab,kw OR (smart headband):ti,ab,kw OR (Google glasses):ti,ab,kw OR (Limb-wn devices):ti,ab,kw OR (wearable clothing):ti,ab,kw OR (electronic skin):ti,ab,kw OR (head-wn devices):ti,ab,kw  #2 Search: (neoplas*):ti,ab,kw OR (oncolog*):ti,ab,kw OR (cancer*):ti,ab,kw OR (tumor*):ti,ab,kw OR (tumour*):ti,ab,kw OR (carcinom*):ti,ab,kw OR (melanom*):ti,ab,kw OR (lymphom*):ti,ab,kw OR (leumemi*):ti,ab,kw OR (malignan*):ti,ab,kw OR (metasta*):ti,ab,kw OR (carcinogen*):ti,ab,kw OR (oncogen*):ti,ab,kw OR (anticarcinogen*):ti,ab,kw OR (sarcoma*):ti,ab,kw OR (blastoma*):ti,ab,kw OR (meningioma*):ti,ab,kw OR (lymphangioma*):ti,ab,kw OR (lymphangiomyoma*):ti,ab,kw OR (lymphangiosarcoma*):ti,ab,kw OR (hodgkin disease*):ti,ab,kw OR (plasmacytoma*):ti,ab,kw OR (carcinosarcoma*):ti,ab,kw OR (hepatoblastoma*):ti,ab,kw OR (mesenchymoma*):ti,ab,kw OR (chordoma*):ti,ab,kw OR (germinoma*):ti,ab,kw OR (gonadoblastoma*):ti,ab,kw OR (mesonephroma*):ti,ab,kw OR (teratoma):ti,ab,kw OR (teratocarcinoma*):ti,ab,kw  #3 Search: (internet):ti,ab,kw OR (web):ti,ab,kw OR (internet based):ti,ab,kw OR (internet-based):ti,ab,kw OR (web based):ti,ab,kw OR (web-based):ti,ab,kw OR (webcasts):ti,ab,kw OR (online):ti,ab,kw OR (social media):ti,ab,kw OR (social networking):ti,ab,kw OR (telecommunications):ti,ab,kw OR (telecommunication):ti,ab,kw OR (mobile technology):ti,ab,kw OR (mobile device):ti,ab,kw OR (mobile app):ti,ab,kw OR (phone):ti,ab,kw OR (mobile phone):ti,ab,kw OR (smart phone):ti,ab,kw OR (cell phone):ti,ab,kw OR (celluar phone):ti,ab,kw OR (blog):ti,ab,kw OR (blogging):ti,ab,kw OR (SMS):ti,ab,kw OR (computer):ti,ab,kw OR (tablet):ti,ab,kw OR (tablet computer):ti,ab,kw OR (video):ti,ab,kw OR (video recording):ti,ab,kw OR (video conference):ti,ab,kw OR (video conferencing):ti,ab,kw OR (video meeting):ti,ab,kw OR (interactive video):ti,ab,kw OR (TV meeting):ti,ab,kw OR (webcast):ti,ab,kw OR (ehealth):ti,ab,kw OR (telemedicine):ti,ab,kw OR (telecare):ti,ab,kw OR (telehomecare):ti,ab,kw OR (telerehabilitation):ti,ab,kw OR (telehealth):ti,ab,kw OR (mhealth):ti,ab,kw OR (telemonitor):ti,ab,kw OR (telemanagement):ti,ab,kw OR (mobile health):ti,ab,kw OR (mhealth):ti,ab,kw OR (mcare):ti,ab,kw OR (mobile communication):ti,ab,kw OR (remote consult*):ti,ab,kw OR (e-health):ti,ab,kw OR (tele-medicine):ti,ab,kw OR (tele-care):ti,ab,kw OR (tele-homecare):ti,ab,kw OR (tele-rehabilitation):ti,ab,kw OR (tele-health):ti,ab,kw OR (m-health):ti,ab,kw OR (tele-monitor):ti,ab,kw OR (tele-management):ti,ab,kw OR (m-health):ti,ab,kw OR (m-care):ti,ab,kw  #4 Search: #1 AND #3  #5 Search: #2 AND #4 |
