# Supplementary material for: The Effectiveness of Wearable Electronic Device System–Supported Physical Activity Programs for Cancer Survivors: Meta-Analysis of Randomized Controlled Trials
Source: J Med Internet Res. 2025 Aug 14;27:e74347. doi: 10.2196/74347 (PMC12352708; doi:10.2196/74347)
Supplement: Multimedia Appendix 2 [file jmir-v27-e74347-s002.docx]

**Table 1 Characteristics of included research**

| Author, year | Country | Sample size  (I/C) | Age | Type of cancer | Stage of cancer | Types of WEDs | Content of BCT | Types of partnering tools | Intervention content | Intervention time | Comparison | Types of usual care | Assessment time | Assessment tools | Assessment period |
| --- | --- | --- | --- | --- | --- | --- | --- | --- | --- | --- | --- | --- | --- | --- | --- |
| Alberts et al. 2020 | USA | 63(31/32) | 44.1±8.7 | Non-specific | NA | Spire Stone | Self-monitoring；Feedback and monitoring | Application | Breath exercises | 4 weeks | Wait list | / | 4 weeks | SF-12-physical health | Post-intervention |
| Anderson, A. S. et al. 2018 | UK | 78(39/39) | 47.1±12.8 | Breast or colorectal CA | NA | Pedometer | Self-monitoring; Goal setting; Implementation intention | Telephone calls and websites | Personalised graduated walking plan and guidance on setting personal goals | 12 weeks | Usual care | education | 12 weeks | Sensewear physical activity monitor, PAQSF, BMI | Post-intervention |
| Bennett et al. 2007 | New Zealand | 47(23/24) | 57.8±10.18 | Non-specific | NA | Digi-Walker | Self-monitoring; Goal setting | Telephone calls | moderate-intensity exercise | 24 weeks | Usual care | education | 24 weeks | CHAMPS, SF-36-physical component | Post-intervention |
| Blair, C. K. et al. 2021 | USA | 35(17/18) | 69.6±4.8 | Breast, prostate and a variety of CA | NA | Jawbone UP2 activity tracker | Self-monitoring; Goal setting; Implementation intention; Feedback and monitoring | Telephone calls and application | Education materials and tech support and light-intensity PA | 13 weeks | Wait list | / | 13 weeks | ActiGraph, SF-36-physical function component | Post-intervention |
| Cadmus-Bertram et al. 2019 | USA | 47(24/23) | 54.4±11.18 | Breast and colorectal CA | Stage I–III in colorectal cancer and no limit in breast cancer | Fitbit charge HR/ Fitbit charge 2 | Self-monitoring; Goal setting; Feedback and monitoring; social support | E-mail and application | MVPA | 12 weeks | Usual care | education | 12 weeks | ActiGraph | Post-intervention |
| Chan et al. 2022 | USA | 41(20/21) | 55.0±11.35 | Colorectal CA | NA | Fitbit Flex | Self-monitoring; Goal setting; | SMS | VPA | 12 weeks | Usual care | education | 12 weeks | SF-36-physical component, FACT-G, | Post-intervention |
| Chan et al. 2020 | USA | 78(40/38) | 69.75±2.25 | Colorectal CA | NA | Fitbit Alta | Self-monitoring; Goal setting; Feedback and monitoring | Application and SMS | Aerobic exercise and strength training | 12 weeks | Usual care | education | 12 weeks | CHAMPS | Post-intervention |
| Chow et al. 2021 | USA | 41(24/17) | 41.21±8.65 | Leukemia or lymphoma | NA | Fitbit Flex | Self-monitoring; Goal setting; Feedback and monitoring; Social support | Application and SMS | Physical activity and diet plan | 16 weeks | Usual care | Only access to websites or app | 12 weeks | ActiGraph, PROMIS global | Post-intervention |
| Ferrante et al. 2020 | USA | 35(18/17) | 61.4±8.83 | Breast CA | Stage 0-III | Fitbit charge | Self-monitoring; Goal setting; Feedback and monitoring | Website and SMS | Physical activity and diet plan | 48 weeks | Wait list | / | 48 weeks | Fitbit, QLACS, BMI | Post-intervention |
| Frensham et al. 2018 | Australia | 91(46/45) | 65.65±9.3 | Non-specific | NA | Pedometer | Self-monitoring; Goal setting; Feedback and monitoring | Website | Any types of physical activity | 12 weeks | Wait list | / | 12 weeks | Pedometer, SF-36-physical component | Post-intervention |
| Gehring et al, 2018 | Netherland | 28(9/19) | 48±10.1 | Glioma | NA | Sports watch with heart rates monitoring | Self-monitoring; Goal setting; Feedback and monitoring | Website and telephone | Aerobic exercises | 24 weeks | Wait list | / | 24 weeks | IPAQ, BMI | Post-intervention |
| Golsteijn et al. 2018 | Netherland | 412(208/204) | 66.5±7.6 | Prostate and colorectal cancer | NA | Pedometer | Self-monitoring; Goal setting; Feedback and monitoring | Website | Computer-tailored PA advice | 16 weeks | Usual care | education | 24 weeks | ActiGraph,,SQUASH, QLQ-C30-general | 8 weeks after intervention |
| Hardcastle et al. 2024 | Australia | 61(32/29) | 63.1±11.1 | Breast and colorectal CA | NA | Fitbit charge 2 | Self-monitoring; Goal setting; Feedback and monitoring | Application and Telephone calls | MVPA | 12 weeks | Usual care | education | 12 weeks | ActiGraph | Post-intervention |
| Hardcastle et al. 2021 | Australia | 64(33/31) | 64.07±7.94 | Colorectal or gynecologic cancer | Stage I and II | Fitbit Alta | Self-monitoring; Goal setting; Feedback and monitoring | Telephone calls | MVPA | 12 weeks | Usual care | education | 24 weeks | ActiGraph, BMI, | 12 weeks after intervention |
| Hartman et al. 2018 | USA | 77(43/44) | 57.2±10.4 | Breast CA | NA | Fitbit one | Self-monitoring; Goal setting; Feedback and monitoring | Telephone calls and e-mail | MPA | 12 weeks | Wait list | / | 12 weeks | ActiGraph, BMI | Post-intervention |
| Howell et al. 2018 | USA | 78(53/25) | 12.7±7.87 | Non-specific | NA | Activity monitor | Self-monitoring; Goal setting; Feedback and monitoring | Website | Any types of PA | 12 weeks | Usual care | education | 24 weeks | ActiGraph , PedsQL | 12 weeks after intervention |
| Johnson et al. 2022 | USA | 49(26/23) | 33.6±4.9 | Non-specific | NA | Fitbit Flex | Self-monitoring; Goal setting; Feedback and monitoring; Social support | Application and SMS | Any types of PA | 12 weeks | Usual care | Only access to websites or app | 12 weeks | ActiGraph | Post-intervention |
| Kenfield et al. 2019 | USA | 64(32/32) | 64.96±2.04 | Prostate cancer | stage ≤ T3a | Fitbit one | Self-monitoring; Goal setting; Feedback and monitoring | Website and SMS | Any types of PA | 12 weeks | Usual care | education | 12 weeks | ActiGraph | Post-intervention |
| Ji Young Kim et al. 2019 | Korea | 58(30/28) | 56.2±9.4 | Colorectal cancer | Stage II–III | Pedometer | Self-monitoring; Goal setting; Feedback and monitoring | Telephone calls and SMS | MVPA | 12 weeks | Usual care | Education | 12 weeks | GLTEQ, FACT-C, BMI | Post-intervention |
| Li et al. 2022 | China | 95(47/48) | 29.82±6.05 | Non-specific | NA | Intelligent sports bracelet | Self-monitoring; Goal setting; Feedback and monitoring | Telephone calls and application | Any types of PA | 8 weeks | Usual care | Education | 8 weeks | FACT-G, PSQI-total | Post-intervention |
| Ligibel et al. 2012 | USA | 99(48/51) | 54.29±10.72 | Breast, colon or rectal cancer | stage I–III | Pedometer | Self-monitoring; Goal setting; Feedback and monitoring | Telephone calls | MVPA | 16 weeks | Usual care | education | 16 weeks | 7-DAYPAR, QLQ-C30-general, BMI | Post-intervention |
| Lynch et al. 2019 | Australia | 80(40/40) | 61.6±6.4 | Breast CA | Stage I-III | Garmin Vivofit 2 | Self-monitoring; Goal setting; Feedback and monitoring | Telephone calls and application | Any types of PA | 12 weeks | Usual care | / | 12 weeks | ActiGraph | Post-intervention |
| Matthews et al. 2007 | USA | 36(22/14) | 53.5±10.6 | Breast CA | stage I–III | Pedometer | Self-monitoring; Goal setting; Feedback and monitoring | Telephone calls | MPA | 12 weeks | Wait list | / | 12 weeks | ActiGraph, CHAMPS | Post-intervention |
| Maxwell‐Smith et al. 2019 | Australia | 61(32/29) | 64.07±7.94 | Colorectal or endometrial cancer | Stage I - II | Fitbit Alta | Self-monitoring; Goal setting; Feedback and monitoring | Website and telephone calls | MVPA | 12 weeks | Usual care | education | 12 weeks | ActiGraph , BMI | Post-intervention |
| McNeil J et al. 2019 | USA | 42(15/14/13) | 59±8.9 | Breast CA | NA | Polar A360(R) device | Self-monitoring; Goal setting; Feedback and monitoring | Application and telephone | LPA | 12 weeks | Usual care | education | 12 weeks | ActiGraph, FACT-B, SF-12-physical health, BMI | Post-intervention |
| Mendoza et al. 2017 | USA | 59(29/30) | 16.6±1.5 | Non-specific | NA | Fitbit Flex | Self-monitoring; Goal setting; Feedback and monitoring; Social support | Application, telephone calls and SMS | Any types of PA | 10 weeks | Usual care | education | 10 weeks | ActiGraph, PedsQL, | Post-intervention |
| Millstine et al. 2019 | USA | 28(15/13) | 55.85±10.76 | Breast CA | NA | Muse headband | Self-monitoring; Goal setting; Feedback and monitoring | Application | Any types of PA | 12 weeks | Usual care | education | 12 weeks | QLQ-C30-general | Post-intervention |
| Nguyen et al. 2021 | Australia | 61(32/29) | 62±6.4 | Breast CA | NA | Garmin Vivofit2 | Self-monitoring; Goal setting; Feedback and monitoring | Telephone calls | Any types of PA | 12 weeks | Wait list |  | 12 weeks | BMI, PSQI-total | Post-intervention |
| Park et al. 2021 | Korea | 148(75/73) | 66.4±7.5 | Prostate CA | NA | Neofit band | Self-monitoring; Goal setting; Feedback and monitoring | Application | Personalized exercise programs | 12 weeks | Usual care | education | 12 weeks | IPAQ, QLQ-C30-general, QLQ-PR25, | Post-intervention |
| Phillips et al. | USA | 49(25/24) | 54.8±11.3 | Breast CA | NA | Fitbit | goal-setting, social support, self-efficacy | Application, telephone calls, | Any types of PA | 12 weeks | Wait list | / | 12 weeks | ActiGraph, FACT-B | Post-intervention |
| Pinto et al. 2005 | USA | 86(43/43) | 53.14±9.7 | Breast CA | Stage 0 to II | Digiwalker | Self-monitoring; Goal setting; Feedback and monitoring | Telephone calls | Any types of PA | 12 weeks | Usual care | education | 12 weeks | 7-DAYPAR, BMI | Post-intervention |
| Pinto(B) et al. 2013 | USA | 192(106/86) | 56±9.87 | Breast CA | NA | Digiwalker | Self-monitoring; Goal setting; Feedback and monitoring | Telephone calls | Any types of PA | 12 weeks | Usual care | education | 12 weeks | CHAMPS, 7-DAYPAR, FACT-C | Post-intervention |
| Pinto(C) et al. 2013 | USA | 43(19/24) | 59.5±11.07 | Colorectal CA | Stage I - III | Digiwalker | Self-monitoring; Goal setting; Feedback and monitoring | Telephone calls | Any types of PA | 12 weeks | Usual care | education | 12 weeks | 7-DAYPAR | Post-intervention |
| Pinto et al. 2015 | USA | 68(36/38) | 55.62±9.55 | Breast CA | Stage 0 - III | Digiwalker | Self-monitoring; Goal setting; Feedback and monitoring | Telephone calls | Any types of PA | 12 weeks | Usual care | education | 12 weeks | CHAMPS, 7-DAYPAR, | Post-intervention |
| Pinto et al. 2021 | USA | 19(11/8) | 71.55±3.97 | Non-specific | Stage I - III | Fitbit charge 2 | Self-monitoring; Goal setting; Feedback and monitoring | E-mail and application | Any types of PA | 12 weeks | Usual care | education | 12 weeks | ActiGraph | Post-intervention |
| Pope et al. 2018 | USA | 20(12/8) | 52.6±9.34 | Breast CA | Stage 0–III | Polar M400 | Self-monitoring; Goal setting; Feedback and monitoring; Social support | Application | Any types of PA | 10 weeks | Usual care | education | 10 weeks | ActiGraph | Post-intervention |
| Rastogi et al. | USA | 50(26/24) | 54.4±11.2 | Breast and colorectal CA | Stage I-III | Fitbit Charge HR or Charge 2 | Self-monitoring; Goal setting; Feedback and monitoring; Social support | Websites+ e-mail | Any types of PA | 12 weeks | Usual care | education | 12 weeks | SF-36 | Post-intervention |
| Sajid et al. 2016 | USA | 11(6/5) | 73.79±7.74 | Prostate cancer | NA | Pedometer | Self-monitoring; Goal setting; Feedback and monitoring | Telephone calls | Aerobic exercise and resistance training | 6 weeks | Usual care | education | 6 weeks | Pedometer, BMI | Post-intervention |
| Uhm et al. 2017 | USA | 339(167/172) | 50.3±9.5 | Breast CA | Stage 0–II | Pedometer | Self-monitoring; Goal setting; Feedback and monitoring | Application | Aerobic and resistance exercises | 12 weeks | Usual care | education | 12 weeks | IPAQ, QLQ-C30-general, QLQ-BR23, BMI, | Post-intervention |
| Vallance et al. 2020 | Canada | 80(40/40) | 62±6.4 | Breast CA | stage I-III | Garmin Vivofit2 | Self-monitoring; Goal setting; Feedback and monitoring | Telephone calls | Any types of PA | 12 weeks | Wait list | / | 12 weeks | FACT-B | Post-intervention |
| Valle et al. 2023 | USA | 280(140/140) | 33.4±4.8 | Non-specific | NA | Fitbit Alta/ Fitbit inspire | Self-monitoring; Goal setting; Feedback and monitoring; Social support | Website, application and SMS | MVPA | 24 weeks | Usual care | education | 24 weeks | ActiGraph, GLTEQ | Post-intervention |
| Van Blarigan et al. 2019 | USA | 39(20/19) | 54±11 | Colorectal CA | stage II-III | Fitbit Flex | Self-monitoring; Goal setting; Feedback and monitoring | Website and SMS | MVPA | 12 weeks | Usual care | education | 12 weeks | ActiGraph, SF-12-physical health, | Post-intervention |
| Van Blarigan et al. 2022 | USA | 44(22/22) | 53.75±4.25 | Colorectal CA | NA | Fitbit Flex | Self-monitoring; Goal setting; Feedback and monitoring | Website and SMS | MVPA | 12 weeks | Usual care | education | 12 weeks | ActiGraph | Post-intervention |
| Van Blarigan et al. 2023 | USA | 51(26/25) | 62.9±6.8 | Prostate Cancer | NA | Polar  H10 heart rate chest strap | Self-monitoring; Goal setting; Feedback and monitoring | Application and telephone calls | Walking | 16 weeks | Usual care | education | 16 weeks | SF-36 | Post-intervention |
| Walsh et al. 2021 | UK | 123(61/62) | 57.36±8.04 | Non-specific | NA | Fitbit Alta | Self-monitoring; Goal setting; Feedback and monitoring | SMS | Any types of PA | 12 weeks | Usual care | education | 12 weeks | Fitbit, GLTEQ, RAND-36-general, BMI | Post-intervention |
| Weiner et al. 2019 | USA | 77(43/44) | 57.19±10.22 | Breast CA | NA | Fitbit one | Self-monitoring; Goal setting; Feedback and monitoring | Telephone calls | MVPA | 12 weeks | Wait list | / | 12 weeks | ActiGraph | Post-intervention |

AAS: [Adult Attachment Scale](http://www.obhrm.net/index.php/Adult_Attachment_Scale_%E6%88%90%E4%BA%BA%E4%BE%9D%E6%81%8B%E9%87%8F%E8%A1%A8%EF%BC%88Fraley%E7%AD%89%EF%BC%8C2000%EF%BC%89); BMI: Body Mass Index; CHAMPS: Community Healthy Activities Model Program for Seniors; EPIC-26: Expanded Prostate Cancer Index Composite- 26; EQ-5DVAS: EuroQoL 5-Dimension and its associated visual analogue scale; FACT-B: [Functional Assessment of Cancer Therapy - B](https://www.facit.org/measures/fact-g)reast cancer; FACT-C: [Functional Assessment of Cancer Therapy - C](https://www.facit.org/measures/fact-g)olorectal; FACT-G: [Functional Assessment of Cancer Therapy - General](https://www.facit.org/measures/fact-g); GLTEQ: Godin Leisure-Time Exercise; IPAQ: [International Physical Activity Questionnaire](https://www.sralab.org/rehabilitation-measures/international-physical-activity-questionnaire-long-form); MPA: Moderate physical activity; MVPA: Moderate-to-vigorous physical activity; PAQSF: Physical Activity Questionnaire Short Form; PedsQL: Pediatric Quality of Life Inventory; PROMIS: Patient-Reported Out-comes Measurement Information System; QLACS: Quality of life in adult cancer survivors; QLQ-BR23: EORTC QLG Core Questionnaire-Breast; QLQ-C30: EORTC QLG Core Questionnaire; QLQ-PR25: EORTC QLG Core Questionnaire-Prostate; RAND-36-general: [RAND-36 Measure of Health-Related Quality of Life](https://www.rand.org/pubs/reprints/RP971.html); SF-12: short form health survey-12-physical health; SF-36: short form health survey-36; SQUASH: [The Short Questionnaire to Assess Health-Enhancing](https://pubmed.ncbi.nlm.nih.gov/26104341/); VPA:Vigorous physical activity; 7-DAY PAR: Seven-Day Physical Activity Recall;
