# Supplementary material for: The Effectiveness of Wearable Electronic Device System–Supported Physical Activity Programs for Cancer Survivors: Meta-Analysis of Randomized Controlled Trials
Source: J Med Internet Res. 2025 Aug 14;27:e74347. doi: 10.2196/74347 (PMC12352708; doi:10.2196/74347)
Supplement: Multimedia Appendix 3 [file jmir-v27-e74347-s003.docx]

| **Outcomes** | **Subgroup** | | **No.of studies** | **No.of Participants** | **Pooled effect size** [95% *CI*] | ***p*-Value for pooled results** | **I^2^** (%) | ***p*-Value for heterogeneity** | **Egger’s test  (*p*-Value)** |
| --- | --- | --- | --- | --- | --- | --- | --- | --- | --- |
| **Objective-measured MVPA** | | | **23** | **1853** | **0.66 [0.47, 0.86]** | ***p*＜0.0001** | **69** | **< 0.01** | **0.2579** |
|  | **Usage of multi-partnering tools** | |  |  |  |  |  |  |  |
|  |  | Yes | 17 | 1110 | 0.68 [0.44, 0.92] | *p*＜0.0001 | 70 | < 0.01 |  |
|  |  | No | 6 | 743 | 0.63 [0.26, 1.01] | *p*＜0.0001 | 71 | < 0.01 |  |
|  | **Duration** | |  |  |  |  |  |  |  |
|  |  | < 12 weeks | 2 | 79 | -0.07 [-0.52, 0.37] | *p*=0.75 | 0 | 0.07 |  |
|  |  | ≥ 12 weeks | 21 | 1774 | 0.72 [0.53, 0.92] | *p*＜0.0001 | 67 | < 0.01 |  |
|  | **Intervention designed for specific cancer type** | | | |  |  |  |  |  |
|  |  | Yes | 18 | 1369 | 0.74 [0.53, 0.96] | *p*＜0.0001 | 64 | < 0.01 |  |
|  |  | No | 5 | 484 | 0.37 [0.04, 0.7] | *p*=0.02 | 52 | 0.08 |  |
| **Subjectively-reported PA** | | | **15** | **2016** | **0.50 [0.23, 0.77]** | ***p*=0.0003** | **79** | **< 0.01** | **0.0876** |
|  | **Usage of multi-partnering tools** | |  |  |  |  |  |  |  |
|  |  | Yes | 4 | 453 | 0.79 [-0.05, 1.63] | *p*=0.06 | 92 | < 0.01 |  |
|  |  | No | 11 | 1563 | 0.39 [0.17, 0.61] | *p*＜0.0001 | 62 | < 0.01 |  |
|  | **Duration** | |  |  |  |  |  |  |  |
|  |  | < 12 weeks | 1 | 19 | -0.00 [-0.91, 0.91] | / | / | / |  |
|  |  | ≥ 12 weeks | 14 | 1997 | 0.52 [0.24, 0.81] | *p*＜0.0001 | 80 | < 0.01 |  |
|  | **Intervention designed for specific cancer type** | | | |  |  |  |  |  |
|  |  | Yes | 12 | 1662 | 0.56 [0.23, 0.89] | *p*＜0.0001 | 82 | < 0.01 |  |
|  |  | No | 3 | 354 | 0.25 [0.04, 0.46] | *P*=0.02 | 0 | 0.48 |  |
| **Steps per day** | | | **16** | **977** | **0.54 [0.14, 0.94]** | ***P*=0.002** | **81** | **< 0.01** | **0.1232** |
|  | **Usage of multi-partnering tools** | |  |  |  |  |  |  |  |
|  |  | Yes | 12 | 787 | 0.59 [0.07, 1.10] | *p*=0.006 | 85 | < 0.01 |  |
|  |  | No | 4 | 190 | 0.32 [-0.17, 0.81] | *p*=0.2 | 46 | 0.14 |  |
|  | **Duration** | |  |  |  |  |  |  |  |
|  |  | < 12 weeks | 2 | 31 | 0.35 [-0.53, 1.23] | *p*=0.44 | 27 | 0.24 |  |
|  |  | ≥ 12 weeks | 14 | 946 | 0.55 [0.11, 0.99] | *p=*0.003 | 83 | < 0.01 |  |
|  | **Intervention signed for specific cancer type** | | | |  |  |  |  |  |
|  |  | Yes | 13 | 554 | 0.59 [0.1, 1.08] | *p*=0.008 | 83 | < 0.01 |  |
|  |  | No | 3 | 423 | 0.17 [-0.02, 0.36] | *p*=0.22 | 43 | 0.17 |  |
| **Sedentary behavior** | |  | **14** | **912** | **-0.63 [-1.34, 0.07]** | ***p*=0.0784** | **92** | **< 0.01** | **0.1549** |
|  | **Usage of multi-partnering tools** | |  |  |  |  |  |  |  |
|  |  | Yes | 12 | 833 | -0.47 [-1.18, 0.24] | *p*=0.06 | 86 | < 0.01 |  |
|  |  | No | 2 | 79 | -1.2 [-3.35, 0.95] | *p*=0.28 | 97 | < 0.01 |  |
|  | **Duration** | |  |  |  |  |  |  |  |
|  |  | < 12 weeks | 3 | 169 | -0.05 [-0.49, 0.40] | *p*=0.83 | 0 | 0.83 |  |
|  |  | ≥ 12 weeks | 11 | 743 | -0.74 [-1.56, 0.08] | *p*=0.0769 | 93 | < 0.01 |  |
|  | **Intervention signed for specific cancer type** | | | |  |  |  |  |  |
|  |  | Yes | 10 | 505 | -0.52 [-1.17, 0.13] | *p*=0.09 | 90 | < 0.01 |  |
|  |  | No | 4 | 407 | -0.95 [-3.12, 1.21] | *p*=0.17 | 95 | < 0.01 |  |
| **BMI** |  |  | **13** | **1169** | **-0.07 [-0.18, 0.05]** | ***p*=0.2675** | **0** | **0.49** | **0.1286** |
|  | **Usage of multi-partnering tools** | |  |  |  |  |  |  |  |
|  |  | Yes | 7 | 320 | -0.23 [-0.53, 0.08] | *p*=0.13 | 34 | 0.17 |  |
|  |  | No | 6 | 849 | -0.02 [-0.15; 0.12] | *p*=0.81 | 0 | 0.99 |  |
|  | **Duration** | |  |  |  |  |  |  |  |
|  |  | < 12 weeks | 0 | 0 | / | / | / | / |  |
|  |  | ≥ 12 weeks | 13 | 1169 | -0.07 [-0.18, 0.05] | *p*=0.27 | / | / |  |
|  | **Intervention signed for specific cancer type** | | | |  |  |  |  |  |
|  |  | Yes | 10 | 895 | -0.01 [-0.15, 0.12] | *p*=0.83 | 0 | 0.97 |  |
|  |  | No | 3 | 274 | -0.29 [-0.74, 0.16] | *p*=0.18 | 67 | 0.05 |  |
| **QoL** |  |  | **21** | **2254** | **0.19 [0.08, 0.31]** | ***p*=0.0008** | **33** | **0.07** | **0.2381** |
|  | **Usage of multi-partnering tools** | |  |  |  |  |  |  |  |
|  |  | Yes | 7 | 397 | 0.35 [0.05, 0.65] | *p*=0.0009 | 56 | 0.03 |  |
|  |  | No | 14 | 1857 | 0.12 [0.03, 0.21] | *p*=0.02 | 0 | 0.62 |  |
|  | **Duration** | |  |  |  |  |  |  |  |
|  |  | < 12 weeks | 3 | 217 | 0.54 [-0.05, 1.12] | *p*=0.08 | 78 | < 0.01 |  |
|  |  | ≥ 12 weeks | 18 | 2037 | 0.12 [0.04, 0.21] | *p=*0.005 | 0 | 0.86 |  |
|  | **Intervention signed for specific cancer type** | | | |  |  |  |  |  |
|  |  | Yes | 14 | 1691 | 0.14 [0.04, 0.23] | *p=*0.006 | 0 | 0.68 |  |
|  |  | No | 7 | 563 | 0.27 [-0.03, 0.56] | *p*=0.08 | 67 | < 0.01 |  |
