# Supplementary material for: The Effectiveness of Wearable Electronic Device System–Supported Physical Activity Programs for Cancer Survivors: Meta-Analysis of Randomized Controlled Trials
Source: J Med Internet Res. 2025 Aug 14;27:e74347. doi: 10.2196/74347 (PMC12352708; doi:10.2196/74347)
Supplement: Multimedia Appendix 4 [file jmir-v27-e74347-s004.docx]

Figure 4-1 Sensitivity analysis of total effects of WEDS-supported PA programs on objectively-measured MVPA[1-21]


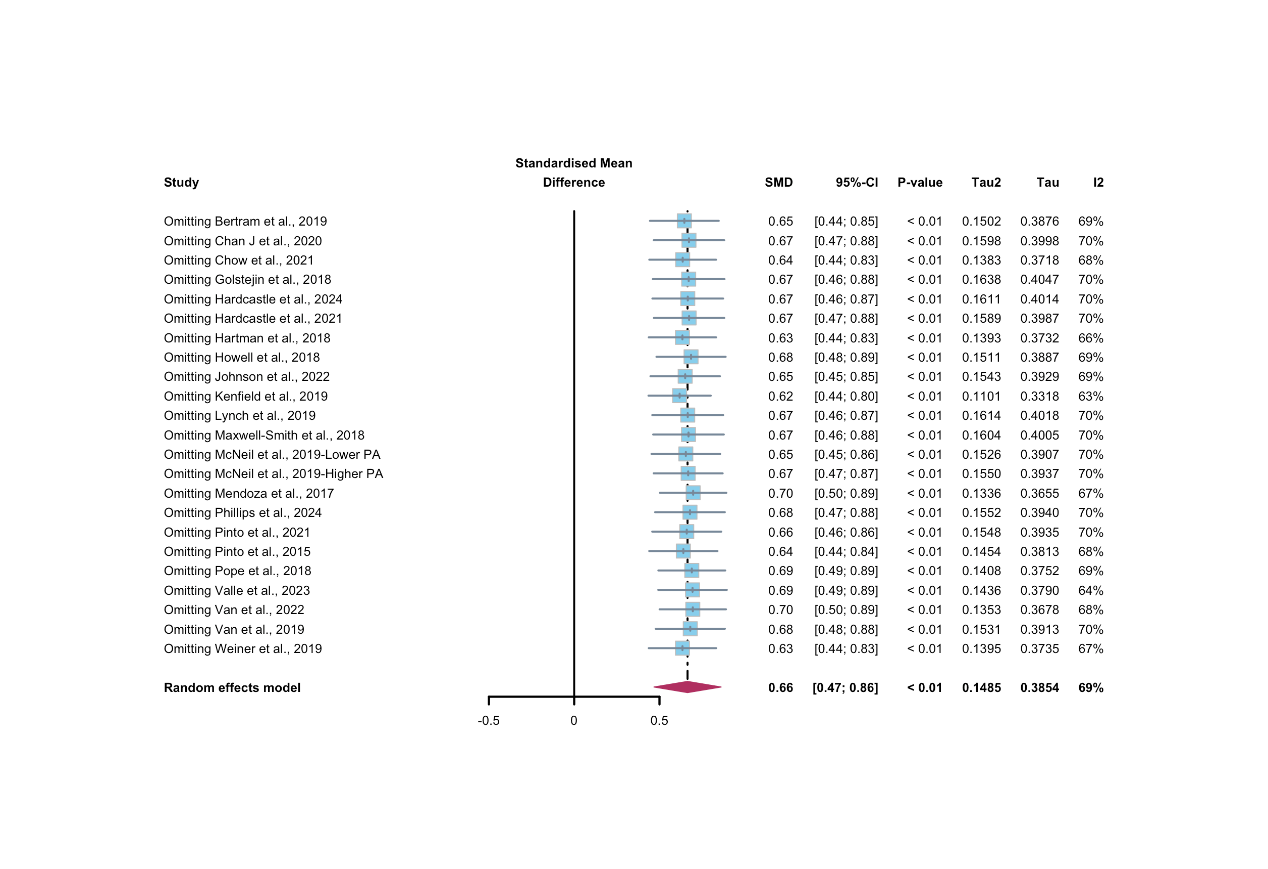


Figure 4-2 Sensitivity analysis of total effects of WEDS-supported PA programs on subjectively-reported PA[2, 4, 18, 22-32]


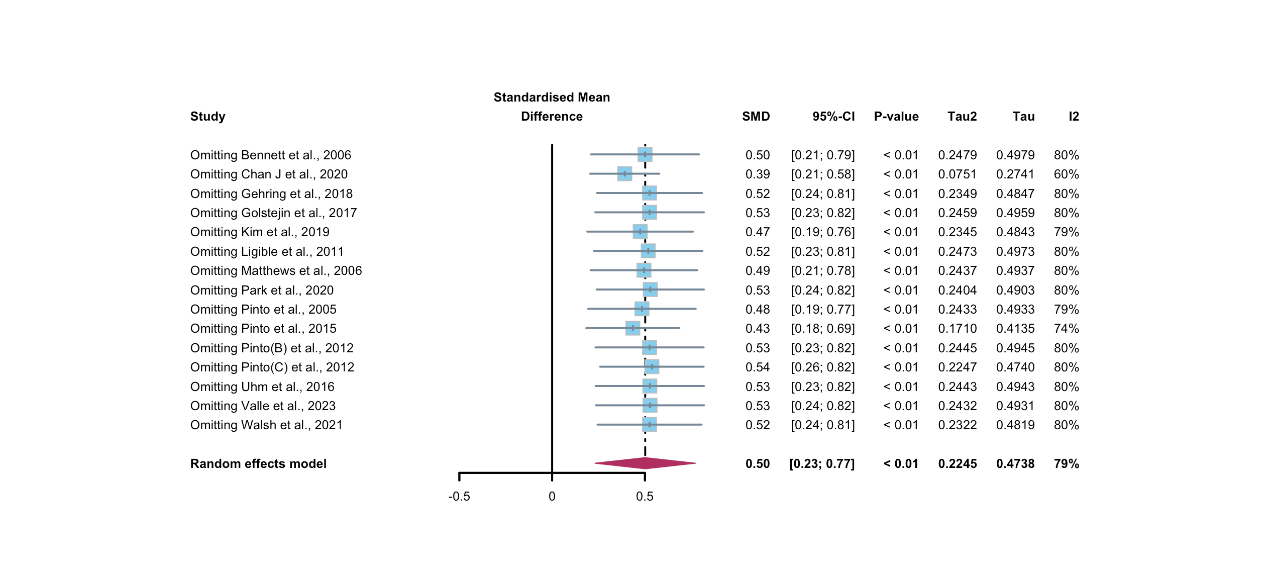


Figure 4-3 Sensitivity analysis of total effects of WEDS-supported PA programs on steps per day[1, 3, 9, 10, 14, 15, 17-20, 26, 32-36]


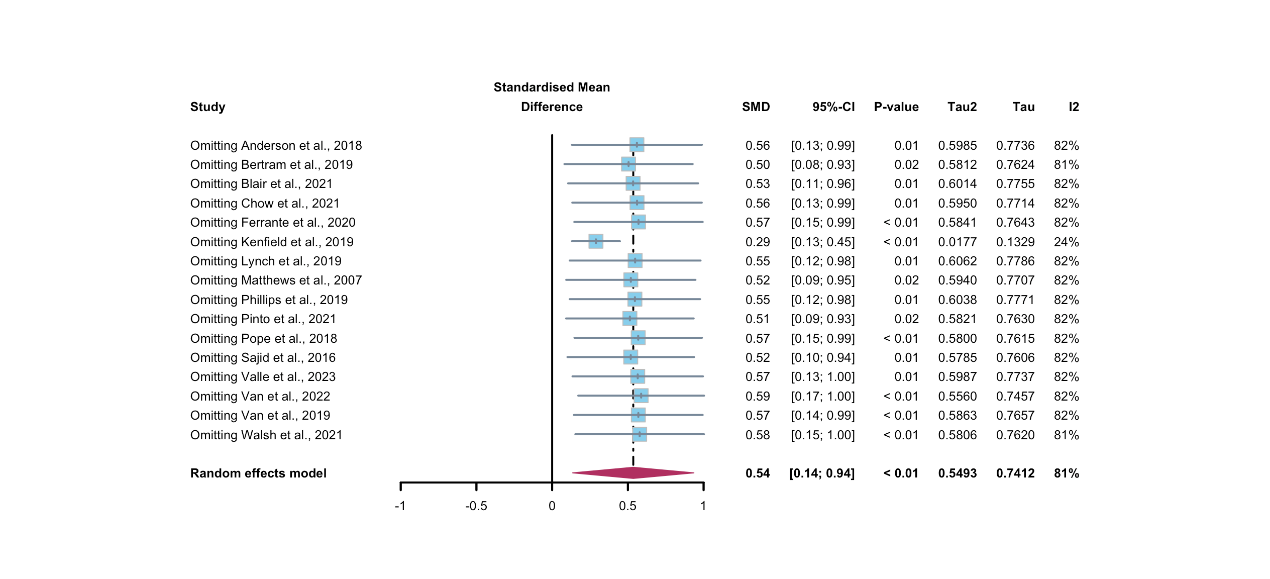


Figure 4-4 Sensitivity analysis of total effects of WEDS-supported PA programs on sedentary behavior[3, 5, 6, 8, 10-13, 15, 17, 18, 34, 37]


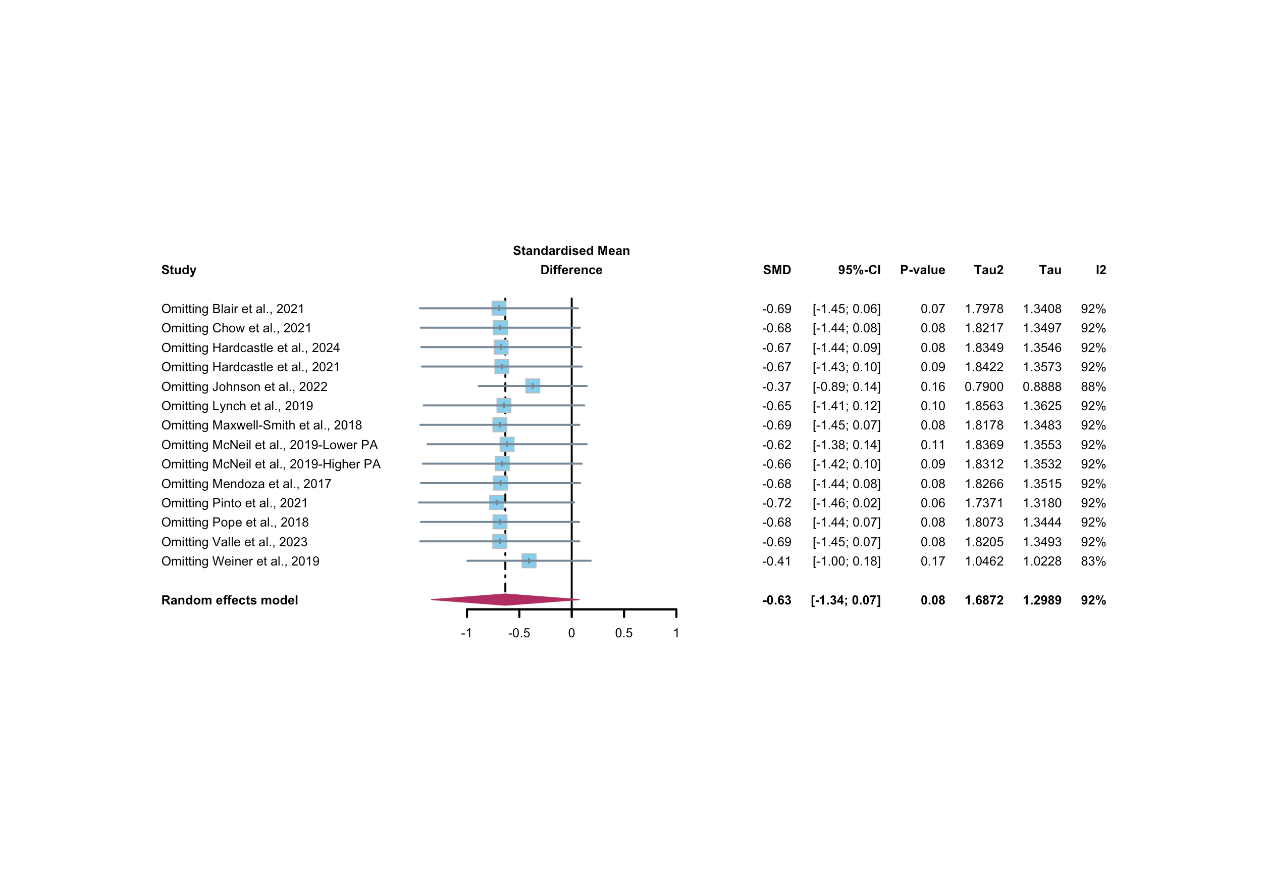
 Figure 4-5 Sensitivity analysis of total effects of WEDS-supported PA programs on BMI[6, 11, 12, 23, 27, 28, 31, 32, 35, 38-40]


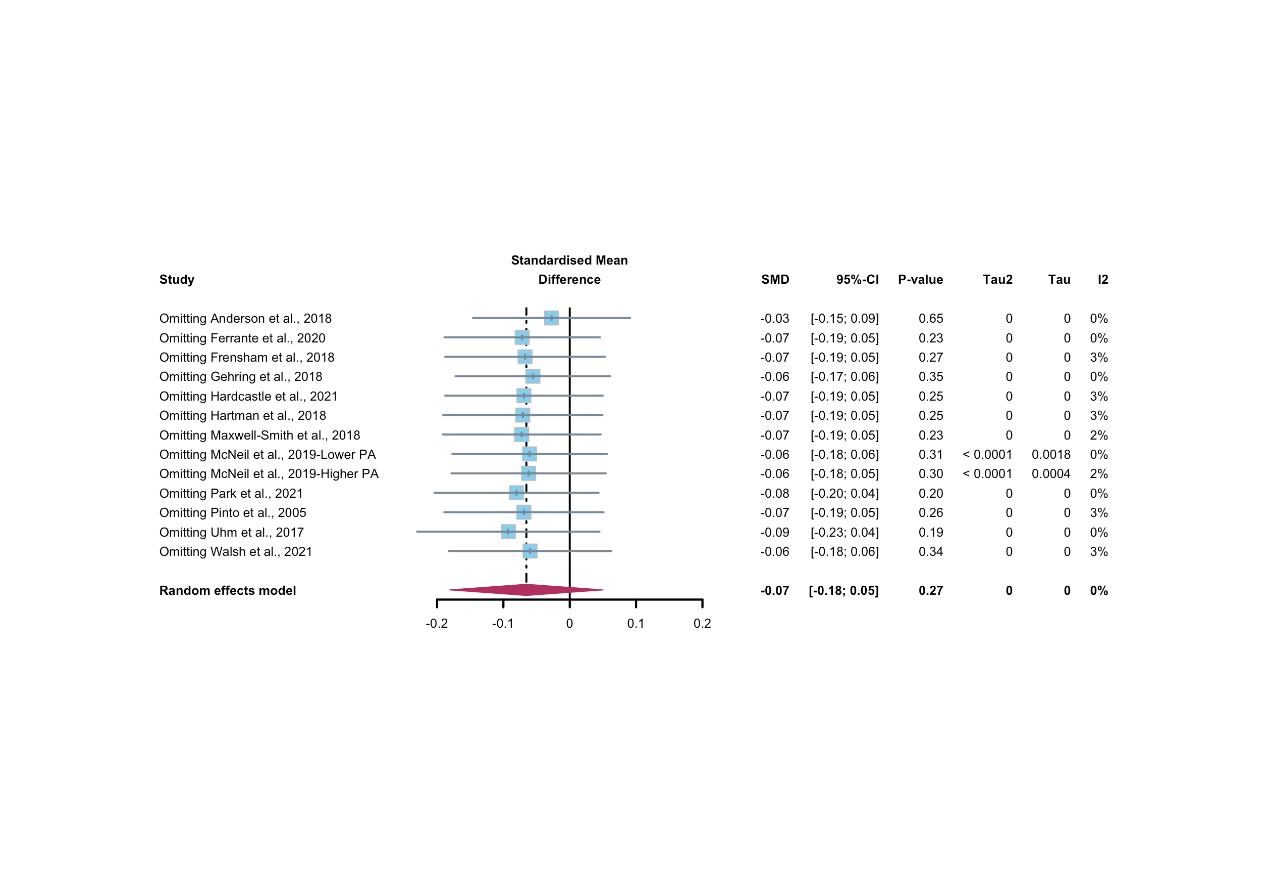


Figure 4-6 Sensitivity analysis of total effects of WEDS-supported PA programs on QoL[3, 4, 7, 13, 14, 22, 24, 25, 27, 29-33, 35, 39, 41-45]


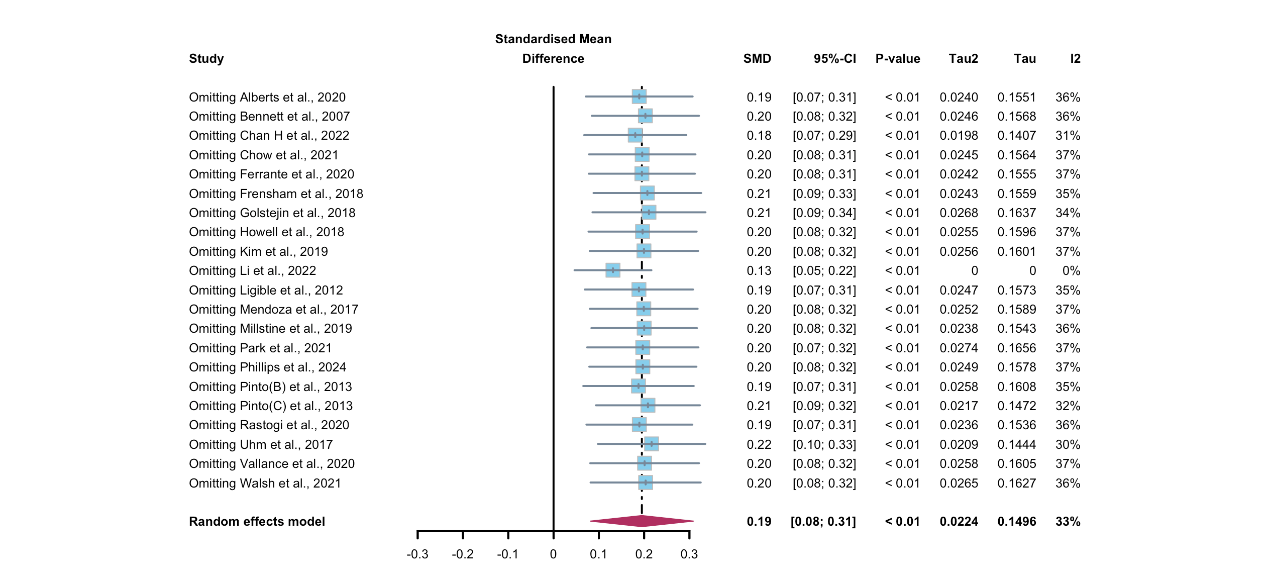


[1] Cadmus-Bertram, L., Tevaarwerk, A. J., Sesto, M. E., Gangnon, R., Van Remortel, B., and Date, P. (2019) Building a physical activity intervention into clinical care for breast and colorectal cancer survivors in Wisconsin: a randomized controlled pilot trial, *J Cancer Surviv* *13*, 593-602.

[2] Chan, J. M., Van Blarigan, E. L., Langlais, C. S., Zhao, S., Ramsdill, J. W., Daniel, K., Macaire, G., Wang, E., Paich, K., Kessler, E. R., Beer, T. M., Lyons, K. S., Broering, J. M., Carroll, P. R., Kenfield, S. A., and Winters-Stone, K. M. (2020) Feasibility and Acceptability of a Remotely Delivered, Web-Based Behavioral Intervention for Men With Prostate Cancer: Four-Arm Randomized Controlled Pilot Trial, *J Med Internet Res* *22*, e19238.

[3] Chow, E. J., Doody, D. R., Di, C., Armenian, S. H., Baker, K. S., Bricker, J. B., Gopal, A. K., Hagen, A. M., Ketterl, T. G., Lee, S. J., and et al. (2021) Feasibility of a behavioral intervention using mobile health applications to reduce cardiovascular risk factors in cancer survivors: a pilot randomized controlled trial, *Journal of cancer survivorship* *15*, 554‐563.

[4] Golsteijn, R. H. J., Bolman, C., Volders, E., Peels, D. A., de Vries, H., and Lechner, L. (2018) Short-term efficacy of a computer-tailored physical activity intervention for prostate and colorectal cancer patients and survivors: a randomized controlled trial, *Int J Behav Nutr Phys Act* *15*, 106.

[5] Hardcastle, S. J., Maxwell-Smith, C., Cavalheri, V., Boyle, T., Román, M. L., Platell, C., Levitt, M., Saunders, C., Sardelic, F., Nightingale, S., McCormick, J., Lynch, C., Cohen, P. A., Bulsara, M., and Hince, D. (2024) A randomized controlled trial of Promoting Physical Activity in Regional and Remote Cancer Survivors (PPARCS), *J Sport Health Sci* *13*, 81-89.

[6] Hardcastle, S. J., Maxwell-Smith, C., Hince, D., Bulsara, M. K., Boyle, T., Tan, P., Levitt, M., Salama, P., Mohan, G., Salfinger, S., Makin, G., Tan, J., Platell, C., and Cohen, P. A. (2021) The wearable activity technology and action-planning trial in cancer survivors: Physical activity maintenance post-intervention, *J Sci Med Sport* *24*, 902-907.

[7] Howell, C. R., Krull, K. R., Partin, R. E., Kadan-Lottick, N. S., Robison, L. L., Hudson, M. M., and Ness, K. K. (2018) Randomized web-based physical activity intervention in adolescent survivors of childhood cancer, *Pediatr Blood Cancer* *65*, e27216.

[8] Johnson, A. M., Baker, K. S., Haviland, M. J., Syrjala, K. L., Abbey-Lambertz, M., Chow, E. J., and Mendoza, J. A. (2022) A Pilot Randomized Controlled Trial of a Fitbit- and Facebook-Based Physical Activity Intervention for Young Adult Cancer Survivors, *J Adolesc Young Adult Oncol* *11*, 379-388.

[9] Kenfield, S. A., Van Blarigan, E. L., Ameli, N., Lavaki, E., Cedars, B., Paciorek, A. T., Monroy, C., Tantum, L. K., Newton, R. U., Signorell, C., Suh, J. H., Zhang, L., Cooperberg, M. R., Carroll, P. R., and Chan, J. M. (2019) Feasibility, Acceptability, and Behavioral Outcomes from a Technology-enhanced Behavioral Change Intervention (Prostate 8): A Pilot Randomized Controlled Trial in Men with Prostate Cancer, *Eur Urol* *75*, 950-958.

[10] Lynch, B. M., Nguyen, N. H., Moore, M. M., Reeves, M. M., Rosenberg, D. E., Boyle, T., Vallance, J. K., Milton, S., Friedenreich, C. M., and English, D. R. (2019) A randomized controlled trial of a wearable technology-based intervention for increasing moderate to vigorous physical activity and reducing sedentary behavior in breast cancer survivors: The ACTIVATE Trial, *Cancer* *125*, 2846-2855.

[11] Maxwell-Smith, C., Hince, D., Cohen, P. A., Bulsara, M. K., Boyle, T., Platell, C., Tan, P., Levitt, M., Salama, P., Tan, J., and et al. (2019) A randomized controlled trial of WATAAP to promote physical activity in colorectal and endometrial cancer survivors, *Psycho-oncology* *28*, 1420‐1429.

[12] McNeil, J., Brenner, D. R., Stone, C. R., O'Reilly, R., Ruan, Y., Vallance, J. K., Courneya, K. S., Thorpe, K. E., Klein, D. J., and Friedenreich, C. M. (2019) Activity Tracker to Prescribe Various Exercise Intensities in Breast Cancer Survivors, *Med Sci Sports Exerc* *51*, 930-940.

[13] Mendoza, J. A., Baker, K. S., Moreno, M. A., Whitlock, K., Abbey-Lambertz, M., Waite, A., Colburn, T., and Chow, E. J. (2017) A Fitbit and Facebook mHealth intervention for promoting physical activity among adolescent and young adult childhood cancer survivors: A pilot study, *Pediatr Blood Cancer* *64*.

[14] Phillips, S. M., Starikovsky, J., Solk, P., Desai, R., Reading, J. M., Hasanaj, K., Wang, S. D., Cullather, E., Lee, J., Song, J., and et al. (2024) Feasibility and preliminary effects of the Fit2ThriveMB pilot physical activity promotion intervention on physical activity and patient reported outcomes in individuals with metastatic breast cancer, *Breast cancer research and treatment* *208*, 391‐403.

[15] Pinto, B. M., Kindred, M., Franco, R., Simmons, V., and Hardin, J. (2021) A 'novel' multi-component approach to promote physical activity among older cancer survivors: a pilot randomized controlled trial, *Acta Oncol* *60*, 968-975.

[16] Pinto, B. M., Stein, K., and Dunsiger, S. (2015) Peers promoting physical activity among breast cancer survivors: A randomized controlled trial, *Health Psychol* *34*, 463-472.

[17] Pope, Z. C., Zeng, N., Zhang, R., Lee, H. Y., and Gao, Z. (2018) Effectiveness of Combined Smartwatch and Social Media Intervention on Breast Cancer Survivor Health Outcomes: A 10-Week Pilot Randomized Trial, *J Clin Med* *7*.

[18] Valle, C. G., Diamond, M. A., Heiling, H. M., Deal, A. M., Hales, D. P., Nezami, B. T., Pinto, B. M., LaRose, J. G., Rini, C. M., and Tate, D. F. (2023) Effect of an mHealth intervention on physical activity outcomes among young adult cancer survivors: The IMPACT randomized controlled trial, *Cancer* *129*, 461-472.

[19] Van Blarigan, E. L., Chan, H., Van Loon, K., Kenfield, S. A., Chan, J. M., Mitchell, E., Zhang, L., Paciorek, A., Joseph, G., Laffan, A., Atreya, C. E., Fukuoka, Y., Miaskowski, C., Meyerhardt, J. A., and Venook, A. P. (2019) Self-monitoring and reminder text messages to increase physical activity in colorectal cancer survivors (Smart Pace): a pilot randomized controlled trial, *BMC Cancer* *19*, 218.

[20] Van Blarigan, E. L., Dhruva, A., Atreya, C. E., Kenfield, S. A., Chan, J. M., Milloy, A., Kim, I., Steiding, P., Laffan, A., Zhang, L., Piawah, S., Fukuoka, Y., Miaskowski, C., Hecht, F. M., Kim, M. O., Venook, A. P., and Van Loon, K. (2022) Feasibility and Acceptability of a Physical Activity Tracker and Text Messages to Promote Physical Activity During Chemotherapy for Colorectal Cancer: Pilot Randomized Controlled Trial (Smart Pace II), *JMIR Cancer* *8*, e31576.

[21] Weiner, L. S., Takemoto, M., Godbole, S., Nelson, S. H., Natarajan, L., Sears, D. D., and Hartman, S. J. (2019) Breast cancer survivors reduce accelerometer-measured sedentary time in an exercise intervention, *J Cancer Surviv* *13*, 468-476.

[22] Bennett, A. (2007) Motivational Interviewing to Increase Physical Activity in Long-Term Cancer Survivors.

[23] Gehring, K., Kloek, C. J., Aaronson, N. K., Janssen, K. W., Jones, L. W., Sitskoorn, M. M., and Stuiver, M. M. (2018) Feasibility of a home-based exercise intervention with remote guidance for patients with stable grade II and III gliomas: a pilot randomized controlled trial, *Clinical rehabilitation* *32*, 352‐366.

[24] Kim, J. Y., Lee, M. K., Lee, D. H., Kang, D. W., Min, J. H., Lee, J. W., Chu, S. H., Cho, M. S., Kim, N. K., and Jeon, J. Y. (2019) Effects of a 12-week home-based exercise program on quality of life, psychological health, and the level of physical activity in colorectal cancer survivors: a randomized controlled trial, *Support Care Cancer* *27*, 2933-2940.

[25] Ligibel, J. A., Meyerhardt, J., Pierce, J. P., Najita, J., Shockro, L., Campbell, N., Newman, V. A., Barbier, L., Hacker, E., Wood, M., Marshall, J., Paskett, E., and Shapiro, C. (2012) Impact of a telephone-based physical activity intervention upon exercise behaviors and fitness in cancer survivors enrolled in a cooperative group setting, *Breast Cancer Res Treat* *132*, 205-213.

[26] Matthews, C. E., Wilcox, S., Hanby, C. L., Der Ananian, C., Heiney, S. P., Gebretsadik, T., and Shintani, A. (2007) Evaluation of a 12-week home-based walking intervention for breast cancer survivors, *Support Care Cancer* *15*, 203-211.

[27] Park, Y. H., Lee, J. I., Lee, J. Y., Cheong, I. Y., Hwang, J. H., Seo, S. I., Lee, K. H., Yoo, J. S., Chung, S. H., and So, Y. (2021) Internet of things-based lifestyle intervention for prostate cancer patients on androgen deprivation therapy: a prospective, multicenter, randomized trial, *Am J Cancer Res* *11*, 5496-5507.

[28] Pinto, B. M., Frierson, G. M., Rabin, C., Trunzo, J. J., and Marcus, B. H. (2005) Home-based physical activity intervention for breast cancer patients, *J Clin Oncol* *23*, 3577-3587.

[29] Pinto, B. M., Papandonatos, G. D., and Goldstein, M. G. (2013) A randomized trial to promote physical activity among breast cancer patients, *Health Psychol* *32*, 616-626.

[30] Pinto, B. M., Papandonatos, G. D., Goldstein, M. G., Marcus, B. H., and Farrell, N. (2013) Home-based physical activity intervention for colorectal cancer survivors, *Psychooncology* *22*, 54-64.

[31] Uhm, K. E., Yoo, J. S., Chung, S. H., Lee, J. D., Lee, I., Kim, J. I., Lee, S. K., Nam, S. J., Park, Y. H., Lee, J. Y., and Hwang, J. H. (2017) Effects of exercise intervention in breast cancer patients: is mobile health (mHealth) with pedometer more effective than conventional program using brochure?, *Breast Cancer Res Treat* *161*, 443-452.

[32] Walsh, J. C., Richmond, J., Mc Sharry, J., Groarke, A., Glynn, L., Kelly, M. G., Harney, O., and Groarke, J. M. (2021) Examining the Impact of an mHealth Behavior Change Intervention With a Brief In-Person Component for Cancer Survivors With Overweight or Obesity: Randomized Controlled Trial, *JMIR Mhealth Uhealth* *9*, e24915.

[33] Alberts, N. M., Leisenring, W. M., Flynn, J. S., Whitton, J., Gibson, T. M., Jibb, L., McDonald, A., Ford, J., Moraveji, N., Dear, B. F., and et al. (2020) Wearable Respiratory Monitoring and Feedback for Chronic Pain in Adult Survivors of Childhood Cancer: a Feasibility Randomized Controlled Trial From the Childhood Cancer Survivor Study, *JCO clinical cancer informatics* *4*, 1014‐1026.

[34] Blair, C. K., Harding, E., Wiggins, C., Kang, H., Schwartz, M., Tarnower, A., Du, R., and Kinney, A. Y. (2021) A Home-Based Mobile Health Intervention to Replace Sedentary Time With Light Physical Activity in Older Cancer Survivors: Randomized Controlled Pilot Trial, *JMIR Cancer* *7*, e18819.

[35] Ferrante, J. M., Devine, K. A., Bator, A., Rodgers, A., Ohman-Strickland, P. A., Bandera, E. V., and Hwang, K. O. (2020) Feasibility and potential efficacy of commercial mHealth/eHealth tools for weight loss in African American breast cancer survivors: pilot randomized controlled trial, *Transl Behav Med* *10*, 938-948.

[36] Sajid, S., Dale, W., Mustian, K., Kotwal, A., Heckler, C., Porto, M., Fung, C., and Mohile, S. G. (2016) Novel physical activity interventions for older patients with prostate cancer on hormone therapy: A pilot randomized study, *J Geriatr Oncol* *7*, 71-80.

[37] Waller, E., Rahman, S., Sutton, P., Allen, J., Saxton, J., and Aziz, O. (2020) Randomised controlled trial of patients undergoing prehabilitation with wearables versus standard of care before major abdominal cancer surgery (Trial Registration: NCT04047524), *Colorectal disease* *22*, 7.

[38] Anderson, A. S., Dunlop, J., Gallant, S., Macleod, M., Miedzybrodzka, Z., Mutrie, N., O'Carroll R, E., Stead, M., Steele, R. J. C., Taylor, R. S., Vinnicombe, S., and Berg, J. (2018) Feasibility study to assess the impact of a lifestyle intervention (a LivingWELL') in people having an assessment of their family history of colorectal or breast cancer, *BMJ Open* *8*, e019410.

[39] Frensham, L. J., Parfitt, G., and Dollman, J. (2018) Effect of a 12-Week Online Walking Intervention on Health and Quality of Life in Cancer Survivors: A Quasi-Randomized Controlled Trial, *Int J Environ Res Public Health* *15*.

[40] Hartman, S. J., Nelson, S. H., Myers, E., Natarajan, L., Sears, D. D., Palmer, B. W., Weiner, L. S., Parker, B. A., and Patterson, R. E. (2018) Randomized controlled trial of increasing physical activity on objectively measured and self-reported cognitive functioning among breast cancer survivors: The memory & motion study, *Cancer* *124*, 192-202.

[41] Chan, H., Van Loon, K., Kenfield, S. A., Chan, J. M., Mitchell, E., Zhang, L., Paciorek, A., Joseph, G., Laffan, A., Atreya, C., Fukuoka, Y., Miaskowski, C., Meyerhardt, J. A., Venook, A. P., and Van Blarigan, E. L. (2022) Quality of life of colorectal cancer survivors participating in a pilot randomized controlled trial of physical activity trackers and daily text messages, *Support Care Cancer* *30*, 4557-4564.

[42] Li, L., Wang, L., Sun, Q., Xiao, P., Duan, Y., Liu, X., Zhou, J., Xie, J., and Cheng, A. S. K. (2022) Effect of Two Interventions on Sleep Quality for Adolescent and Young Adult Cancer Survivors: a Pilot Randomized Controlled Trial, *Cancer nursing* *45*, E560‐E572.

[43] Millstine, D. M., Bhagra, A., Jenkins, S. M., Croghan, I. T., Stan, D. L., Boughey, J. C., Nguyen, M. T., and Pruthi, S. (2019) Use of a Wearable EEG Headband as a Meditation Device for Women With Newly Diagnosed Breast Cancer: A Randomized Controlled Trial, *Integr Cancer Ther* *18*, 1534735419878770.

[44] Rastogi, S., Tevaarwerk, A. J., Sesto, M., Van Remortel, B., Date, P., Gangnon, R., Thraen-Borowski, K., and Cadmus-Bertram, L. (2020) Effect of a technology-supported physical activity intervention on health-related quality of life, sleep, and processes of behavior change in cancer survivors: a randomized controlled trial, *Psycho-oncology* *29*, 1917‐1926.

[45] Vallance, J. K., Nguyen, N. H., Moore, M. M., Reeves, M. M., Rosenberg, D. E., Boyle, T., Milton, S., Friedenreich, C. M., English, D. R., and Lynch, B. M. (2020) Effects of the ACTIVity And TEchnology (ACTIVATE) intervention on health-related quality of life and fatigue outcomes in breast cancer survivors, *Psychooncology* *29*, 204-211.
